# Genetic Characterization and Population Structure of Drug-Resistant *Mycobacterium tuberculosis* Isolated from Brazilian Patients Using Whole-Genome Sequencing

**DOI:** 10.3390/antibiotics13060496

**Published:** 2024-05-28

**Authors:** Leonardo Souza Esteves, Lia Lima Gomes, Daniela Brites, Fátima Cristina Onofre Fandinho, Marcela Bhering, Márcia Aparecida da Silva Pereira, Emilyn Costa Conceição, Richard Salvato, Bianca Porphirio da Costa, Reginalda Ferreira de Melo Medeiros, Paulo Cesar de Souza Caldas, Paulo Redner, Margareth Pretti Dalcolmo, Vegard Eldholm, Sebastien Gagneux, Maria Lucia Rossetti, Afrânio Lineu Kritski, Philip Noel Suffys

**Affiliations:** 1 Programa Acadêmico de Tuberculose da Faculdade de Medicina, Centro de Ciências da Saúde, Universidade Federal do Rio de Janeiro (UFRJ), Rio de Janeiro 21941-590, RJ, Brazil; kritskia@gmail.com; 2 Centro de Desenvolvimento Científico e Tecnológico (CDCT), Secretaria Estadual de Saúde (SES-RS), Porto Alegre 90450-190, RS, Brazil; richardsalvato@hotmail.com; 3 Laboratório de Biologia Molecular Aplicado à Micobactérias, Fundação Oswaldo Cruz (FIOCRUZ), Instituto Oswaldo Cruz (IOC), Rio de Janeiro 21040-360, RJ, Brazil; lialima2212@gmail.com (L.L.G.); psuffys@gmail.com (P.N.S.); 4 Swiss Tropical and Public Health Institute (Swiss TPH), CH-4123 Allschwil, Switzerland; d.brites@swisstph.ch (D.B.); sebastien.gagneux@swisstph.ch (S.G.); 5 University of Basel, CH-4001 Basel, Switzerland; 6 Centro de Referência Professor Hélio Fraga, Fundação Oswaldo Cruz (FIOCRUZ), Rio de Janeiro 22780-195, RJ, Brazil; fatima.fandinho@fiocruz.br (F.C.O.F.); marcelabhering@yahoo.com.br (M.B.); marcapas2011@gmail.com (M.A.d.S.P.); bporphirio@yahoo.com.br (B.P.d.C.); regimedeiros@hotmail.com (R.F.d.M.M.); paulocesarcaldas@ensp.fiocruz.br (P.C.d.S.C.); predner@gmail.com (P.R.); margarethdalcolmo@ensp.fiocruz.br (M.P.D.); 7 Department of Science and Innovation—National Research Foundation Centre of Excellence for Biomedical Tuberculosis Research, South African Medical Research Council Centre for Tuberculosis Research, Division of Molecular Biology and Human Genetics, Faculty of Medicine and Health Sciences, Stellenbosch University, Cape Town 7505, South Africa; emilyncosta@gmail.com; 8 Norwegian Institute of Public Health, 0213 Oslo, Norway; v.eldholm@gmail.com; 9 Laboratório de Biologia Molecular, Universidade Luterana do Brasil (ULBRA), Canoas 92425-020, RS, Brazil; mrossett@terra.com.br

**Keywords:** *Mycobacterium tuberculosis*, drug resistance, whole-genome sequencing, genetic diversity, Brazil, novel mutations

## Abstract

The present study aimed to determine the genetic diversity of isolates of *Mycobacterium tuberculosis* (Mtb) from presumed drug-resistant tuberculosis patients from several states of Brazil. The isolates had been submitted to conventional drug susceptibility testing for first- and second-line drugs. Multidrug-resistant (MDR-TB) (54.8%) was the most frequent phenotypic resistance profile, in addition to an important high frequency of pre-extensive resistance (p-XDR-TB) (9.2%). Using whole-genome sequencing (WGS), we characterized 298 Mtb isolates from Brazil. Besides the analysis of genotype distribution and possible correlations between molecular and clinical data, we determined the performance of an in-house WGS pipeline with other online pipelines for Mtb lineages and drug resistance profile definitions. Sub-lineage 4.3 (52%) was the most frequent genotype, and the genomic approach revealed a p-XDR-TB level of 22.5%. We detected twenty novel mutations in three resistance genes, and six of these were observed in eight phenotypically resistant isolates. A cluster analysis of 170 isolates showed that 43.5% of the TB patients belonged to 24 genomic clusters, suggesting considerable ongoing transmission of DR-TB, including two interstate transmissions. The in-house WGS pipeline showed the best overall performance in drug resistance prediction, presenting the best accuracy values for five of the nine drugs tested. Significant associations were observed between suffering from fatal disease and genotypic p-XDR-TB (*p* = 0.03) and either phenotypic (*p* = 0.006) or genotypic (*p* = 0.0007) ethambutol resistance. The use of WGS analysis improved our understanding of the population structure of MTBC in Brazil and the genetic and clinical data correlations and demonstrated its utility for surveillance efforts regarding the spread of DR-TB, hopefully helping to avoid the emergence of even more resistant strains and to reduce TB incidence and mortality rates.

## 1. Introduction

Tuberculosis (TB) accounted for 1.4 million deaths and an estimated 10.6 million new cases in 2023, and was the leading cause of death from a single infectious agent worldwide until the COVID-19 pandemic [1]. The emergence of drug-resistant (DR) TB is a global threat that hinders successful TB treatment. Multidrug-resistant TB (MDR-TB), defined as the simultaneous resistance to rifampicin (RIF) and isoniazid (INH), results in a worse prognosis, prolonged TB treatment with second-line drugs that are more toxic, more expensive, and that could possibly evolve into pre-extensive drug-resistant TB (p-XDR-TB). The latter is defined as strains that fulfill the definition of multidrug-resistant or rifampicin-resistant (MDR/RR-TB), plus resistance to any fluoroquinolone, and subsequently can evolve into extensive drug-resistant TB (XDR-TB), defined as strains p-XDR-TB plus resistance to bedaquiline and/or linezolid.

Brazil is one of the thirty countries with the highest TB burden, and although the incidence rate decreased until 2014, during the period of 2015–2019, a notified increased incidence of 34.3 to 37.4 cases/100.000 inhabitants was observed [2]. In addition, due to the COVID-19 pandemic, it is estimated that after a decade of decline, TB mortality has increased in Brazil and globally [3]. An additional concern for TB control in Brazil is the considerable number of DR-TB patients; in 2018, around 2.500 MDR/RR-TB cases were estimated [4], including an increase in MDR-TB among patients that had not previously been treated in the Rio de Janeiro State [5].

Rapid DR-TB detection and epidemiological surveillance, as well as knowledge about the genetic diversity of isolates of the *Mycobacterium tuberculosis* complex (MTBC) in different settings, are factors that may contribute to DR-TB elimination. In this scenario, molecular tools have become important, and quite recently, next-generation sequencing (NGS) has made it possible to quickly characterize the whole genome of MTBC strains, enabling the identification of both resistance-related genetic variants and lineages involved in ongoing transmission [6,7]. The culture-based phenotypic drug susceptibility test (DST), although still the current gold standard, has limitations due to the slow growth rate of MTBC organisms. Thus, molecular methods for drug resistance prediction are being steadily introduced as a routine in low-TB-incidence countries [8].

WGS is a promising tool and an approach to DR/MDR-TB detection, since it provides detailed sequence information from different genomic regions, thus enabling drug resistance prediction [9]. However, the high amount of sequencing data generated by WGS has created the challenge to develop bioinformatics tools to translate the data into information of clinical and laboratory interest [8]. To permit the use of WGS by professionals with little or no bioinformatics skills, user-friendly tools for the analysis and interpretation of WGS data have been developed and implemented, permitting the accessibility and broad implementation of NGS-based approaches [10,11,12,13]. Nonetheless, due to the complexity of large-scale data analyses, some bioinformatics command-line skills are still required and sometimes, a user-friendly graphical interface is not available [14,15].

Due to the global variability in the prevalence of MTBC lineages and the evidence of a differential association with drug resistance, it is evident that the combined detection of both characteristics in different countries and regions may interfere with DR-TB management [16]. Therefore, the genomic information of MTBC strains and a conventional phenotypic DST regarding clinical outcomes are urgently needed [17,18,19]. In addition, the evaluation of the different available tools for the extraction of a DST profile from WGS has become important to evaluating the regional differences in DR-TB surveillance and to understanding local TB transmission [20,21,22,23].

Here we conducted a genetic diversity study on genomes from a large collection of Mtb isolates from several states of Brazil that mostly had a phenotypic DST for primary and secondary drugs. WGS data were evaluated by an in-house WGS pipeline and different online available pipelines, including Mykrobe Predictor, KvarQ, TB Profiler version 0.3.4, and the more recent TB Profiler 5.0 to predict drug susceptibility, and the in-house WGS pipeline, KvarQ, TB Profiler 5.0, and RD-Analyzer to predict the genotype (TB lineages).

## 2. Results

### 2.1. Study Samples, Patient Data, and Phenotypic Drug Susceptibility Testing

Among the 298 Mtb isolates from presumed DR-TB patients with high-quality WGS data, 294 had conventional phenotypic DST data for at least one drug. The phenotypic DST resulted in 64 (21.8%) pan-susceptible, 22 (7.5%) isoniazid-monoresistant (IMR), 9 (3.1%) rifampicin-monoresistant (RMR); 1 (0.3%) ethambutol-monoresistant (EMR), 10 (3.4%) poly-resistant (Poly-R), 161 (54.8%) MDR, and 27 (9.2%) p-XDR (Supplementary Table S1).

Among the 170 patients for which we had identification, 112 had sociodemographic, clinical, radiographic, and treatment outcome data available (Supplementary Table S2). Among them, 65 (57.5%) were male, 7 (6.3%) were smokers, 8 (7.1%) were illicit drug users, 16 (14.2%) had diabetes, 12 (10.7%) were alcohol users, and 7 (6.3%) were HIV-infected. In relation to chest radiographic images, 72 patients (64.3%) had bilateral cavitary disease, 15 (13.4%) had unilateral, and 16 (14.3%) had no image available. Regarding treatment outcomes, 50 (44.6%) were cured or completed treatment, 32 (28.6%) died due to TB, 16 (14.3%) were lost to follow-up, 5 (4.5%) were still under treatment, 5 (4.5%) died from another cause, and 4 (3.6%) patients had no clinical data available.

### 2.2. Novel Mutations, In Silico Drug Susceptibility, and Resistance Prediction Using Different Pipelines

**Figure 1:**
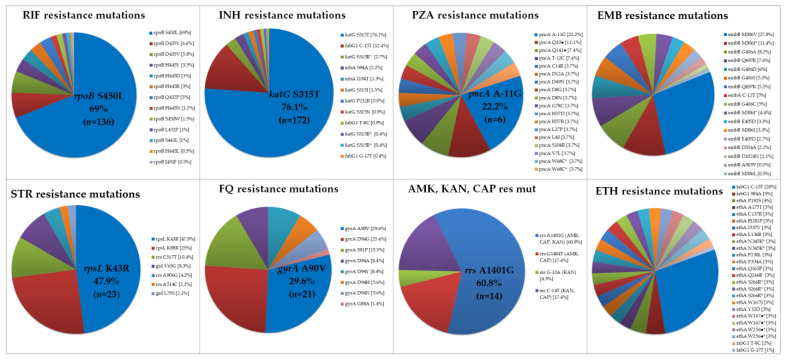
Proportion of resistance-related mutations of 298 isolates included in the study based on in-house WGS pipeline analysis. The most frequent mutation found is highlighted. RIF = rifampicin; INH = isoniazid; PZA = pyrazinamide; EMB = ethambutol; STR = streptomycin; FQ = fluoroquinolone; AMK, KAN, CAP res mut = amikacin, kanamycin, capreomycin resistance mutation; ETH = ethionamide. * = same amino acid changed but different mutation involved. ● = stop codon.

Considering the in-house WGS pipeline, the 298 sequenced genomes were classified as follows: 80 (26.8%) pan-susceptible, 11 (3.7%) IMR, 4 (1.3%) RMR, 1 (0.3%) EMR, 1 (0.3%) SMR, 10 (3.4%) Poly-R, 124 (41.6%) MDR, and 67 (22.5%) p-XDR. The nature and frequencies of the mutations detected with this pipeline are presented in Figure 1 and in Supplementary Table S3.

Using the 294 genomes that had a DST available for at least one drug, we compared the performance in drug resistance using five predictive drug resistance pipelines against the phenotypic DST (three different pipelines and two versions of TB Profiler). The in-house WGS pipeline showed slightly better overall results when considering sensitivity, specificity, positive and negative predictive values, and accuracy compared to Mykrobe Predictor, both versions of TB Profiler, and KvarQ (Table 1). For the prediction of resistance to RIF and INH, in all tools, the sensitivity was higher than 80% but the specificity was lower than 95%. Regarding the prediction of other first-line drugs, the in-house WGS pipeline showed sensitivity higher than 80% to EMB, while high specificity to STR was observed using the in-house WGS pipeline and Mykrobe Predictor. With respect to second-line drugs, the highest sensitivity to FQ ofloxacin (OFL) was obtained using TB Profiler, and over 90% specificity to AMK, KAN, and CAP was observed in all pipelines. The highest sensitivity to PZA was obtained in the most updated version of TB Profiler (68%).

The agreement among phenotypic DST and the in-house WGS pipeline, KvarQ, Mykrobe Predictor, TB Profiler 0.3.4, and TB Profiler 5.0 was assessed for all drugs considering resistance and susceptibility prediction (regardless of mutation type in pipelines). The highest agreement level among the six methods was found in RIF (k = 0.89), and the worst agreement level was in PZA (k = 0.32). An excellent level of agreement was also detected in INH, AMK, KAN, and CAP (Supplementary Table S4).

Using TB Profiler 5.0, which is able to detect and report unique mutations, i.e., those not yet reported, twenty novel mutations in three resistance genes (*katG*, *pncA*, and *ethA*) were identified, of which six were present in eight phenotypically resistant isolates (gene–drug-related), as shown in Table 2. Unfortunately, ethionamide susceptibility was not phenotypically characterized in the present study. Mostly isolates with novel mutations were phenotypically MDR (68.7%) and genotypically p-XDR (46.8%). All mutations observed using TB Profiler 5.0 are shown in Supplementary Table S5.

### 2.3. Genomic Diversity, Phylogenetic Analysis, and Lineage Classification Using Different Pipelines

Among the 298 genomes analyzed by the in-house WGS pipeline, 99% belonged to lineage 4, of which 155 (52%) belonged to sub-lineage 4.3 [LAM], 42 (14.1%) to sub-lineage 4.10 [PGG3], 40 (13.4%) to sub-lineage 4.1.2 [Haarlem], 35 (11.7%) to sub-lineage 4.1.1 [X], 17 (5.7%) to sub-lineage 4.4 [Vietnam], 2 (0.6%) had not been sub-lineage-detected and 4 (1.3%) demonstrated a mixed classification of L4.3/L4.1.1 (n = 1), L4.3/L4.1.2 (n = 1), L4.3/L4.10 (n = 1), and L4.1.1/L4.4 (n = 1), indicating mixed infections or contaminations; three (1%) were classified as lineage 1.

**Figure 2:**
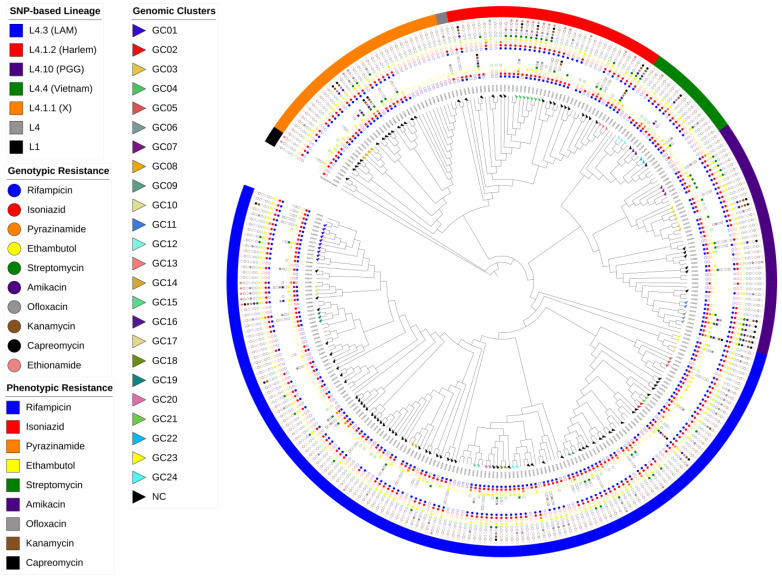
Maximum likelihood phylogenetic tree for the 293 *M. tuberculosis* isolates constructed based on 38,563 SNPs rooted with *M. canettii*. Presented from the outside to the inside: SNP-based lineage classification by the in-house WGS pipeline; genotypic resistance profile (circle); phenotypic resistance profile (square). Tips (triangle) are shown colored according to the genomic cluster from 170 patients with identification (see legend). GC = genomic cluster; NC = non-clustered. Tree branch scale represents the number of nucleotide substitutions per site.

A phylogenetic tree (Figure 2) based on 38,563 genome-wide SNPs was constructed using 293 genomes. Five genomes with a high count of mixed SNP calls were excluded (four with mixed sub-lineage classification and one L4.1.2 [Haarlem]). The topology was congruent with different lineage and sub-lineage classifications assigned by all SNP-based pipelines as previously described [24,25].

Upon comparing the SNP-based lineage classification pipelines (in-house WGS pipeline, KvarQ-barcode-Coll14, and TB Profiler) and an RD-based analysis pipeline (RD-Analyzer), the L4.3 [LAM] (and higher resolutions classifications when available, e.g., L4.3.2, L4.3.3, L4.3.4, L4.3.4.1, L4.3.4.2, L4.3.4.2.1) was predominant with all tools. The classification results and lineage proportions are presented in Supplementary Figure S1.

Some discordant genotypic classifications were encountered and described in Supplementary Figure S2, mainly among RD-based pipeline analyses and SNP-based pipelines. Nevertheless, the free marginal kappa coefficient obtained presented an excellent agreement level (k = 0.89 [95%CI: 0.86–0.93]), and the overall agreement was 92.06%.

**Figure 3:**
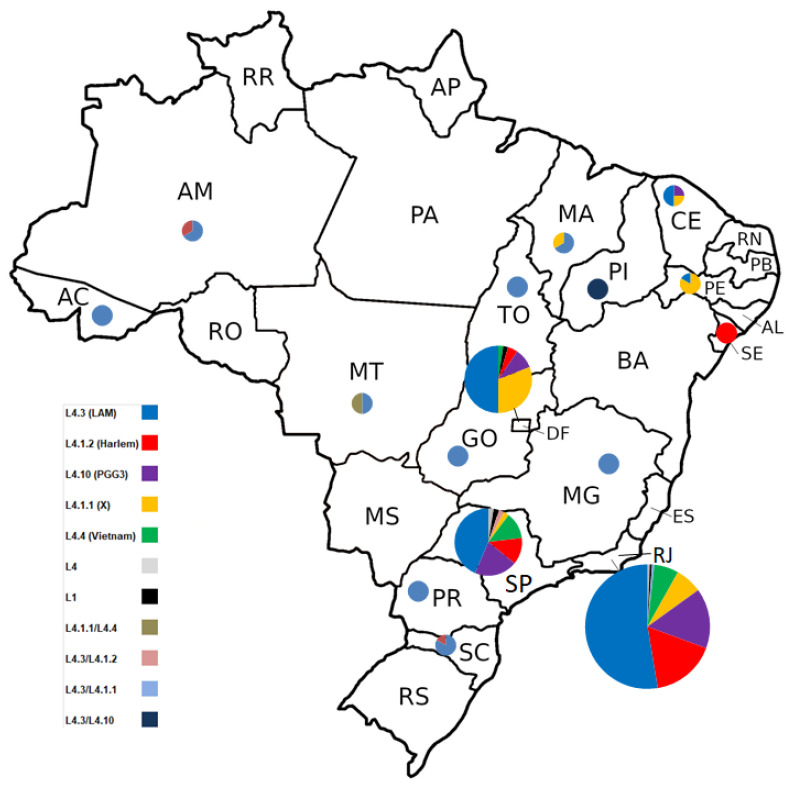
Geographic proportion distribution of *Mycobacterium tuberculosis* lineages classified by in-house WGS pipeline of 290 isolates. Eight isolates (three L4.3 [LAM], two L4.10 [PGG3], two L4.1.1 [X], and one L4.1.2 [Haarlem]) have no data regarding geographic origin.

With respect to the sample origin (Figure 3), 212 (71.2%) were isolates from patients residing in the southeast region, including 173 (58.1%) isolates from Rio de Janeiro and 39 (13.1%) from São Paulo; 46 (15.4%) were from the Midwest region, including 42 (14.1%) from the Distrito Federal, 2 (0.7%) from Goiás, and 2 (0.7%) from Mato Grosso; 15 (4.9%) were form the northeast, including 6 (2%) from Pernambuco, 4 (1.3%) from Ceará, 3 (1%) from Maranhão, 1 (0.3%) from Piauí, and 1 (0.3%) from Sergipe; 9 (3%) were from the south region, including 6 (2%) from Santa Catarina and 3 (1%) from Paraná; finally, 6 (2%) were from the north region, including 3 (1%) from Amazonas, 2 (0.7%) from Tocantins, and one (0.3%) from Acre. For eight (2.7%) isolates, we had no data regarding state origin.

### 2.4. Genomic Clusters Analysis

**Figure 4:**
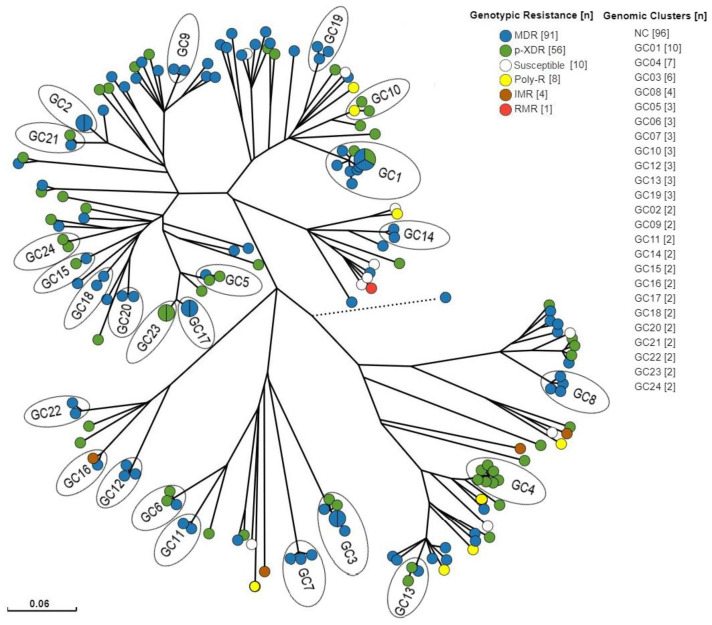
Radial tree of the 170 isolates used for clustering analysis. Tips were colored according to genotypic resistance profile. Pie charts in the tips represent two or more isolates with no SNP differences among genomes. NC = non-clustered. The dashed line represents the L1 isolate with greater distance in comparison to the rest of the isolates. Tree branch scale represents the number of nucleotide substitutions per site. [n] = number of isolates by category.

Among the 170 isolates with patient identification available, 74 (43.5%) belonged to 24 genomic clusters with sizes ranging from 2 to 10 isolates, therefore characterized by a clustering rate of 0.294. The most frequent genotypic resistance profile in this sampling was MDR-TB (53.5%), followed by p-XDR-TB (32.9%), and a similar distribution was observed among the clustered population, composed of 63.5% MDR-TB and 33.7% p-XDR-TB isolates (Figure 4). Importantly, a significant association between being part of a cluster and having an MDR-TB genotype was observed (Chi-squared = 5.2512; *p* = 0.02).

Upon analysis of the genotypes of these 170 isolates using the in-house WGS pipeline, 95 (55.8%) belonged to sub-lineage 4.3 [LAM], 26 (15.2%) were 4.1.2 [Haarlem], 20 (11.7%) were 4.10 [PGG3], 18 (10.5%) were 4.1.1 [X], 9 (5.3%) were 4.4 [Vietnam], 1 (0.6%) was classified as lineage 1, and 1 (0.6%) as lineage 4 without sub-lineage detection. Among the isolates in clusters, L4.3 LAM was also observed most frequently (n = 39; 52.7%).

When concentrating on the origins of these samples, 138 were from Rio de Janeiro (81.1%), 6 each from Pernambuco (3.5%) and Santa Catarina (3.5%), 4 from Ceará (2.3%), 3 each from Amazonas (1.7%), Maranhão (1.7%), and Paraná (1.7%), 2 each from Minas Gerais (1.1%) and Goiás (1.1%), and 1 each from Tocantins (0.6%), Acre (0.6%), and Mato Grosso (0.6%). An even more pronounced number of the clustered isolates (95.9%) were from Rio de Janeiro, while only three isolates were from other states (Ceará [GC8], Goiás, and Tocantins [GC15]). The latter explains why we observed two genomic clusters with isolates belonging to patients from different states: GC8 with one from Ceará and three from Rio de Janeiro, and GC15 with one from Goiás and one from Tocantins.

### 2.5. Evolution of Drug Susceptibility Patterns in Patients with Multiple Isolates of Mycobacterium tuberculosis

Among the 112 patients for whom clinical and epidemiologic data were available, 15 had at least 2 isolates with genomes sequenced, resulting in a total of 36 genomes: 2 patients with 4 isolates, 2 patients with 3 isolates, and 11 patients with 2 isolates each, who could have been included in follow-up (Table 3 and Supplementary Table S6). Only two patients presented a change in phenotypical drug resistance: One was susceptible to AMK and changed to being resistant without a change in mutational profile (patient P39). Another was phenotypically susceptible to KAN and CAP and changed to being resistant (patient P64), again without a change in mutational profile.

Regarding the mutational profile changes of those 15 patients, 7 patients (2 with 3 isolates each and 5 with 2 isolates each) had their genotypic drug resistance profile changed (Table 3). The other eight patients presented no change in the genomes of their respective isolates (genetic distance = 0 SNPs) (Supplementary Table S6).

The emergence of resistance mutations for FQ was observed in five patients in the *gyrA* gene (including P28 = S91P [T→C], P27 = A90V [C→T], P63 = D94H [G→C], P10 = D94A [A→C], and P39 = D94G [A→G]/A90V [C→T]); one patient was cured from the disease while the other four died of tuberculosis. Two patients had an EMB mutation resistance emergence in the *embB* gene (P54 = G406D [G→A] and P35 = Q497R [A→G]); the first was cured while the second patient abandoned treatment with no further information obtained.

### 2.6. Treatment Outcome, Risk Factors, and Lineage Associations with Genotypic and Phenotypic Resistance

The association between TB treatment outcome and genotypic and phenotypic drug resistance was evaluated. Genotypic p-XDR-TB was significantly associated with mortality (Chi-squared = 4.6231; *p* = 0.03). Both phenotypic resistance (Chi-squared = 7.3445; *p* = 0.006) and genotypic resistance to EMB (Fisher’s exact test *p* = 0.0007) were significantly associated with mortality. Another significant association was EMB genotypic resistance (Chi-squared = 24.364; *p* = 0.00001) with the p-XDR-TB genotype. All isolates with a phenotypic EMB available are summarized in Supplementary Table S7. In short, the table shows the available EMB tests of isolates, divided into four groups: EMB genotypically susceptible and resistant and phenotypically susceptible and resistant; each of these groups was also divided into two groups: cured and deceased.

Compared to patients who had any risk factors for developing TB (HIV, diabetes, alcoholism, smoking, and/or illicit drugs abuse) (Supplementary Table S2), among the two outcome groups, deceased and cured, a significant association between the presence of any risk factor and a deceased outcome was observed (Chi-squared = 5.7672; *p* = 0.01).

Upon comparing of the frequency of the presence of drug-resistance-related mutations in Mtb isolates classified as L4.3 (LAM) to those of other sub-lineages, a statistically significantly lower frequency of mutations related to resistance to STR (Chi-squared = 5.3496; *p* = 0.02), CAP (Chi-squared = 6.4498; *p* = 0.01), ETH (Chi-squared = 5.7636; *p* = 0.01), and AMK (Fisher’s exact test *p* = 0.001) was associated with the LAM sub-lineage in comparison to non-LAM isolates.

## 3. Discussion

In this study, we performed a genome-based characterization of the genetic diversity of mainly drug-resistant isolates of Mtb in different regions in Brazil. The analyzed Mtb strains predominantly belonged to the sub-lineage 4.3 (LAM) (52%). This is in accordance with previous studies performed in various Brazilian regions in both susceptible and DR-TB samples [26,27,28,29]. L4.3 was predominant in the south, north, southeast and midwest regions. In the northeast, this predominance of L4.3 was not observed, but the number of samples from this region was low. Three isolates (from São Paulo, Rio de Janeiro, and Distrito Federal) belonged to lineage 1, which are known to be restricted mostly to eastern Africa and the south and east of Asia [30], but are apparently described with a certain frequency in the northern region of Brazil [29,31,32], probably imported trough slave trade from east Africa [23]. Although L1 is usually not associated with DR, two of the three isolates in this study presented some resistance (one IMR and one Poly-R). This might be due to sampling bias as the CRPHF mainly receives samples from patients suspicious of being DR, either due to treatment failure, treatment abandonment, relapse TB, or contact with TB-resistant patients.

The four pipelines presented a high agreement level (k = 0.89), with almost complete agreement among the SNP-based pipelines, but showing divergences mainly in the lineage classifications by RD-Analyzer. The Mtb isolates with high counts of mixed SNP calls may be indicative of a mixed infection, as described by others [33,34,35]. An analysis of the five isolates with a high proportion of mixed SNP calls showed four mixed classifications by the in-house WGS pipeline only, while TB Profiler and KvarQ detected only one of the two lineages present in these isolates. RD-Analyzer had some difficulties in classifying lineage in comparison with the SNP-based tools and presented the most divergences among all the tools tested, including 31 isolates with a mixed classification and 6 unidentified despite having a good genome quality and low mixed SNP call counts.

The majority of clusters were identified in Rio de Janeiro, and although sampling from this state was clearly over-represented in our setting, this may represent higher levels of recent infection and drug-resistant TB transmission, differing from TB cases due to the reactivation of a latent infection [36,37]. In this Brazilian state, an increase in primary MDR-TB has been described as being associated with low TB control performance [5]. Studies in other Brazilian states have also shown significant ongoing transmission of MDR-TB, as observed in Sao Paulo [38,39] and Santa Catarina [40], and Rio Grande do Sul in a genome-based study [41]. In the latter, as well as in our study, a significant association between clustering and genotypic MDR-TB and significant ongoing transmission in p-XDR-TB resistance profiles was observed, with practically half of the patients infected with genotypic p-XDR-TB (44.6%; n = 25) in clusters, reflecting an alarming scenario of insufficient DR-TB control [41].

In addition to clusters of isolates derived from residents of particular states, we also observed clusters suggestive of the interstate transmission of particular genotypes that seem to have been circulating in the country for a considerable time. Examples are GC8 (three patients from Rio de Janeiro and one from Ceará) and GC15 (one from Goiás and one from Tocantins) presenting genetic distances of ≤12 SNPs. Furthermore, we observed among the isolates of unidentified patients, which were not included in clustering analysis, one isolate from Distrito Federal (isolated in the year 2007) that had a genetic distance of ≤12 SNPs to the GC4 isolates that were from Rio de Janeiro (2008–2012), and one isolate from São Paulo (2004) and one isolate from Distrito Federal (2007) that had genetic a distance of 11 SNPs between them. These could point to other two interstate transmission events, requiring further investigation.

From 15 patients, we had several isolates collected during and after treatment. In most of these cases, the infecting lineage strain during follow-up with the patients was the same, and reinfection with another strain and lineage was only observed in one patient (P35). In a retrospective cohort study from China [42], a MIRU-VNTR-based recurrence definition observed a lower frequency of reinfection (n = 21; 36.2%) in comparison to relapse (n = 37; 63.8%). In a population-based study from Malawi [43] using WGS, a lower frequency of reinfection (n = 20; 14.3%) compared with relapse (n = 55; 39.5%) was also observed; 64 (46%) of recurrent patients remained unclassified, showing how challenging it remains to classify such cases.

We observed the emergence of FQ-resistance-conferring mutations among one third of the patients that were followed-up with during treatment. These bacterial populations had likely been selected due non-compliance of drug treatment [44,45]. In addition, the emergence of phenotypic resistance to AMK in patient P39 and phenotypic resistance to KAN and CAP in patient P64 was observed, but without the detection of known mutations in *rrs* and *eis*. This could be caused by not yet known mutations conferring resistance to those drugs and/or by efflux pump activity, not investigated here. Such pumps have been described to be induced by sub-inhibitory levels of antibiotics such as FQ, RIF, and INH in inappropriate treatment regimens [46]. Additionally, the presence of a minor undetected population of drug-resistant bacteria, so-called heteroresistance, can also explain differences between culture and in silico-based drug susceptibility outcomes [47].

Another observation was the emergence of the G406D mutation in *embB* in patient P54 (Table 3); however, a mixed call was detected in this codon and may indicate the selection of the mutant population within a single strain, probably due to inappropriate treatment. Patient P35 presented the emergence of the Q497R mutation in *embB*, and in addition a shift in the RIF resistance genotype, initially presenting the Q432P mutation in *rpoB* and posteriorly the S450L mutation. Besides that, in the first isolate the sub-lineage detected was L4.1.1 (X) and in the second was L4.3 (LAM), and a genomic difference of 484 SNPs was observed between both, indicating a TB recurrence by reinfection [43].

We observed a statistically significantly lower frequency of mutations related to resistance to STR, CAP, ETH, and AMK in strains belonging to sub-lineage 4.3 (LAM) in comparison with non-LAM sub-lineages. A lower frequency of mutations related to resistance in some lineages has been described, including a lower frequency of S315T mutations in *katG* in the LAM spoligofamily when compared to Haarlem [48]. Another study showed a higher mutation rate in Mtb strains of lineage 2 and as such, a faster emergence of resistance-conferring mutations in strains of lineage 2, accompanied by the development of DR-TB [49].

Upon comparing patient treatment outcomes, risk factors for TB development, phenotypic and genotypic resistance, and Mtb lineage, we observed a significant association between EMB phenotypic (Chi-squared *p* = 0.006) and genotypic (Fisher’s exact test *p* = 0.0007) resistance and TB mortality. EMB resistance has been described as a risk factor for mortality, mainly when associated with MDR pattern [50]. In our study, genotypic p-XDR-TB was significantly associated with EMB genotypic resistance, while the genotypic p-XDR-TB profile was significantly associated with a higher morbidity (Chi-squared *p* = 0.03). In addition, the presence of any of the five risk factors (HIV, diabetes, smoking cigarette, alcohol, and/or illicit drug use) was also significantly associated with morbidity but independent of genotypic or phenotypic EMB resistance. The increased risk of mortality in MDR-TB and p-XDR-TB patients has been described [51,52] and is related to the increasing level of drug resistance, whereas accumulating even more factors increases the probability of an unfavorable outcome [53,54]. EMB resistance could be an important stage of resistance accumulation in Mtb.

We observed a similar proportion of main drug-resistance-conferring mutations, frequencies, and affected genes detected by all the pipelines tested, and this is in agreement with other studies [55,56,57,58,59].

Unprecedented was the detection of 20 novel mutations in 32 strains using TB Profiler 5.0, including a novel frameshift mutation *katG*_c.2070delC in an isolate that is likely associated with INH resistance, considering the impact this may have on the catalase-peroxidase structure and activity, accompanied by phenotypic resistance against INH. The same isolate also presented the mutation *ahpC*_c.-81C>T, classified in the WHO catalogue as having uncertain significance, but which may be acting in synergy with the newly described mutation to confer resistance; this needs to be better investigated. This isolate was misclassified as genetically susceptible to INH by all pipelines except TB Profiler, and the same was observed in other isolates with the mutation *ahpC*_c.-81C>T, highlighting the importance of using updated pipelines.

Only 4 of 32 isolates were fully phenotypically characterized for the 9 drugs available in this study. Unfortunately, 13 of the isolates in which we detected a novel mutation were not phenotypically characterized for the drug of interest, making it impossible to establish the genetic and phenotypic correlation. Among the four isolates with the frameshift mutation *pncA*_c.193_200dupTCCTCGTC, two had no PZA DST available and two were PZA-resistant (these two were from the same patient); the mutation *pncA*_c.305dupC was observed in one resistant and one with a DST not available; finally, the mutations *pncA*_c.452dupT and *pncA*_c.75_79delCGCGC were both presents in resistant isolates, all suggesting a resistance association. On the other hand, the frameshift mutation *pncA*_c.443_444dupGC was detected in both susceptible and resistant isolates, making it difficult to interpret. Other phenotypically susceptible isolates observed with novel frameshift mutations in the *pncA* and *katG* genes could be explained by an acquired low-level resistance, under the antibiotic concentration threshold used here or due to some laboratorial error in phenotypic resistance determination. Phenotypically susceptible isolates harboring confident resistance mutations have been reported in other studies [60,61].

Among ten other mutations we detected that were absent in the WHO catalogue (Supplementary Table S5), eight [62,63,64,65,66,67,68,69] in twelve isolates had not yet been reported in Brazil, and four (*katG*_p.Leu634Phe, *pncA*_p.Asp63His, *pncA*_p.Gly23Val, and *pncA*_c.521_522insT) were present in phenotypically resistant isolates in our study. Another mutation associated with resistance was detected for the first time in Brazil, *rpoB*_p.Ile491Phe, a mutation outside of the 81 bp hotspot of the *rpoB* gene, which makes it undetectable by commercial assays such as GeneXpert MTB/RIF and MTBDR*plus* [70,71]. In Eswatini, for example, the presence of this mutation is a significant problem because of its prevalence of >60% in MDR strains [72]. In our study, this mutation was observed in one isolate that was genotypically and phenotypically MDR and carried the *rpoB*_p.Ser493Leu mutation, an uncertain variant according to the WHO. However, their combination could be acting in synergy because the former is a borderline resistance mutation [73].

The comparison of the pipelines, after evaluating their performance in drug resistance prediction through sensitivity, specificity, accuracy, and positive and negative predictive value calculation, using phenotypic resistance results as a reference, showed a better overall performance in resistance prediction by the in-house WGS pipeline. For all pipelines, the sensitivity of the prediction of resistance to RIF and INH was higher than 80% but the specificity was lower than 95%, and therefore below that of the WHO recommendations [74]. A high positive predictive value (PPV) of >80% was observed in all pipelines used for the detection of mutations conferring resistance to RIF, INH, OFL, AMK, and similar to that described by others [20,75]. A lower PPV was observed for the detection of resistance mutations whose action mechanisms are more complex and for which the genetic bases of resistance are less understood, such as resistance to PZA, KAN, and CAP. Unreliable PZA resistance prediction has been reported in various studies [20,31,75,76], and in order to enhance PZA resistance prediction, the following strategies have been described: (i) improving the mutation library of the pipelines mainly by including the detection of indels in *pncA*; (ii) the “non-wild-type sequence” approach, which consists of interpreting any non-synonymous mutations or indels in *pncA* as genotypic PZA-resistant strains; (iii) manually checking the sequence reads [77,78]. Not surprisingly, the best sensitivity performance for PZA resistance prediction was achieved by TB Profiler 5.0, the most up-to-date pipeline for the detection of DR-associated mutations, accounting for the largest PZA mutation catalogue among all pipelines used and able to detect novel mutations, including fourteen novel indels observed in this study.

Another important finding in our study is that, compared to conventional DST, more than twice as many cases were classified as p-XDR-TB using WGS. This difference in proportion could be beyond the accuracy of genomic tests for second-line resistance detection. However, one should take into account that the criteria for performing phenotypic DST tests for second-line drugs in Brazil cause the underestimation of the detection of resistance to such drugs. These results highlight the importance of using WGS for the epidemiological surveillance and control of DR/MDR/p-XDR-TB, as these discrepancies would not be detected and reported without it use.

Without any doubt, a major limitation of this study is that phenotypic tests for second-line drugs were not conducted for all samples. Among the 161 isolates classified as phenotypically MDR-TB, only 22 were tested for all second-line drugs, and 10 were only tested for 9 drugs; 1 of the 10 Poly-R-TB and 6 of the 27 p-XDR-TB were submitted to the full DST. This further emphasizes the importance of using molecular methods for TB diagnosis and comprehensive resistance analyses. Another limitation is the small sample size from regions outside Rio de Janeiro, Distrito Federal, and Sao Paulo States, not representing the national scenario of DR-TB in Brazil.

In conclusion, the evaluated pipelines for the prediction of MTBC lineage and drug resistance work well in the Brazilian sample studied here, and our data favor the use of WGS in cases without a conventional DST. Although the in-house WGS pipeline performed slightly better in general, all tools performed well in predicting DR to RIF, INH, AMK, KAN, and CAP. Importantly they allowed for the detection of p-XDR-TB strains that otherwise would probably have been unreported. The analysis of isolates of DR-TB patients that were sampled sometimes years apart demonstrated that several accumulated drug resistance mutations, showing that resistance evolution occurs by the acquisition of mutations in the same strain, probably due to the non-compliance of treatment. Phylogenetic analysis and lineage characterization contribute to better understanding the MTBC genotypes spreading in the population, so WGS improves the knowledge of MTBC population structure and evolution and offers the rapid and reliable assessment of resistance-related mutations, allowing for faster access to effective treatment. A surprisingly low level of reinfection was observed in an area with high TB incidence, even in patients with DR and prolonged treatment. We believe that these findings highlight the importance of the need for active surveillance throughout the national territory in order to avoid further aggravation of the TB scenario in Brazil.

## 4. Materials and Methods

### 4.1. Sample Collection and Phenotypic Drug Resistance

We used a convenience sample for this study of 298 Mtb clinical isolates (Brazilian SISGEN code: AD20DC4), available at the National Tuberculosis Reference Center Professor Hélio Fraga (CRPHF; Rio de Janeiro, Brazil). The sample set included isolates of patients from 16 different Brazilian states with presumed DR-TB due to a history of treatment failure, treatment abandonment, relapse TB, or contact with DR-TB patients. The isolates were therefore submitted to phenotypic drug susceptibility testing (DST).

DST was performed for the first-line drugs RIF, INH, PZA, and EMB, and to the second-line drugs STR, OFL, AMK, KAN, and CAP, using the liquid MB/BacT system (Organon Teknika Corp., Durham, NC, USA). The following cut-off values were used: RIF (1.0 mg/L), INH (0.1 mg/L), EMB (5.0 mg/L), PZA (100.0 mg/L), STR (1.0 mg/L), OFL (2.0 mg/L), AMK (1.0 mg/L), KAN (4.0 mg/L), and CAP (2.5 mg/L).

### 4.2. Whole-Genome Sequencing and In-House Pipeline

The Mtb genomic DNA were extracted as described previously [79]. To perform WGS, sequencing libraries were prepared as described previously [80], and sequenced on an Illumina platform at Division of Infectious Diseases and Environmental Health of the Norwegian Institute of Public Health. The sequence reads generated were deposited in the Sequence Read Archive (SRA) of the NCBI under the Bioproject PRJEB27366. To describe the MTBC genetic diversity and genotypic resistance profile, a single-nucleotide-polymorphism (SNP)-based Mtb lineage and sub-lineage and resistance-related genes (11 coding genes and four promoter regions) were assigned, first using an in-house pipeline developed at the Swiss Tropical and Public Health Institute (Swiss TPH) as described before [81], hereafter called the in-house WGS pipeline. In brief, FASTQ files were processed with Trimmmomatic v 0.33 (SLIDINGWINDOW:5:20) [82] to remove Illumina adaptors and trim low quality reads. Overlapping reads were then merged with SeqPrep v 1.2. Duplicated reads were marked by the MarkDuplicates module of Picard v 2.9.1. The resulting reads were mapped to a reconstructed ancestral sequence of MTBC as described previously [83] using the BWA-MEM v 0.7.13 algorithm. Pysam v 0.9.0 was used to exclude reads with an alignment score lower than (0.93*read_length)-(read_length*4*0.07)); this corresponds to more than seven mismatches per 100 bp. SNPs were called with Samtools v 1.2 mpileup and VarScan v 2.4.1. The SNP annotation used was that of *Mycobacterium tuberculosis* H37Rv reference strain (NC_000962.3). Repetitive regions of the genome as PE/PPE/PGRS were excluded as described before [84].

### 4.3. Phylogenetic Analysis

A maximum likelihood phylogenetic tree using 100 bootstraps was constructed using an alignment containing all variable positions from all quality filtered genomes with ≥15× average coverage using MEGAX v 10.2 [85], and the resulting tree was rooted using *M. canettii* (Genbank accession number: NC_019950.1). The tree was visualized using iTOL v 6.7 [86].

### 4.4. Lineage Classification Pipelines Comparisons

Mtb lineage classification by the in-house WGS pipeline was compared with three online available pipelines: TB Profiler version 5.0 [21], KvarQ version 0.12.2 [10] but using the SNP barcode used elsewhere [11], and RD-Analyzer version 1.01, a region-of-difference-based analyzer of Mtb from sequence reads [87].

### 4.5. Genotypic Resistance Detection and Pipelines Evaluation

In order to identify the drug-resistance-related variants among the included Mtb genomes, we used the in-house WGS pipeline and the command-line versions of the pipelines Mykrobe Predictor version 0.3.3 [12], TB Profiler version 0.3.4 [76], KvarQ version 0.12.2 [10], and TB Profiler version 5.0 (https://github.com/jodyphelan/TBProfiler, accessed on 28 September 2023), a recent pipeline version that accounts for the updated WHO catalogue of mutations conferring resistance (https://www.who.int/publications/i/item/9789240082410, accessed on 29 January 2024).

### 4.6. Cluster Analysis and Recent Transmission

Genomic clusters were delineated using the tool snp-dists v0.7.0 (https://github.com/tseemann/snp-dists, accessed on 5 August 2022). An SNP-based distance matrix was built using the alignment of 170 high-quality genomes not presenting mixed calls. A genomic cluster was defined when two or more isolates presented genomes with ≤12 SNPs of difference, which encompasses not only linked cases such as household contacts (genetic distance from zero to five SNPs), but also related cases (five to twelve SNPs). These thresholds are considered as very likely representing recent transmission events, especially in settings using isolates from chronically infected patients and DR-TB [88,89,90]. To estimate recent transmission, 170 genomes, corresponding to one genome per patient appropriately identified (patient ID), were used. Samples without patient identification and/or duplicated ID were excluded from this analysis. The reconstructed maximum likelihood tree using 100 bootstraps was visualized in GrapeTree [91]. Clustering rate was calculated using the following formula (nc − c)/n, where nc = the total isolates in cluster, c = the number of clusters, and n = the total number of isolates.

### 4.7. Patient Data

The available patient clinical data, including TB treatment outcome, diabetes, alcohol use, illicit drug use, cigarette smoking, HIV coinfection status, and sex, were obtained from the “Sistema de Informação de Agravos de Notificação” (SINAN) and “Sistema de Informação de Tratamentos Especiais de Tuberculose” (SITETB), two national databases for disease surveillance. The project was approved by the Research Ethics Committee of Federal University of Rio de Janeiro (CAEE 10126919.2.0000.5257).

### 4.8. Statistical Analyses

For the prediction of drug resistance, we calculated sensitivity, specificity, accuracy, and positive and negative predictive values using the statistical software R (Version 3.6.1). First- and second-line anti-TB drug predictions were evaluated for the four pipelines, using MB/BacT DST as the reference standard.

Free marginal Kappa Randolph’s statistics [92] with a 95% confidence interval was used to determine agreement among Mtb lineage classification by the four pipelines. The same procedure was followed to determine agreement among phenotypic drug resistance detected by MB/BacT and the genotypic drug resistance detected by the four pipelines, using four levels of agreement: <0.40 (poor), 0.40–0.59 (fair), 0.60–0.80 (moderate/good) and >0.80 (excellent).

Pearson’s Chi-squared test was used to determine associations among treatment outcome, risk factors, clusters, lineage, genotypic and phenotypic drug resistance using the chisq.test function in the statistical software R (Version 3.6.1). In case the expected value is less than 5, Fisher’s exact test was calculated using the fisher.test function in R.

## Figures and Tables

**Table 1:** Evaluation of WGS-based drug susceptibility prediction based on the in-house WGS pipeline, KvarQ, Mykrobe Predictor, TB Profiler, and TB Profiler 5.0 presenting sensitivity, specificity, positive and negative predictive values, and accuracy.

DST			WGS Pipeline					KvarQ					Mykrobe Predictor					TB Profiler					TB Profiler 5.0				
ATB	RES	SUS	SEN	SPE	PPV	NPV	ACC	SEN	SPE	PPV	NPV	ACC	SEN	SPE	PPV	NPV	ACC	SEN	SPE	PPV	NPV	ACC	SEN	SPE	PPV	NPV	ACC
RIF ¹	199	95	89.95%	84.21%	92.27%	80%	88.1%	88.94%	84.21%	92.19%	78.43%	87.41%	89.95%	84.21%	92.27%	80%	88.1%	87.94%	82.11%	91.15%	76.47%	86.01%	89.95%	83.15%	91.79%	79.79%	87.75%
INH ¹	217	75	85.25%	88%	95.36%	67.35%	85.96%	83.87%	88%	95.29%	65.35%	84.93%	84.79%	88%	95.34%	66.67%	85.62%	88.94%	82.67%	93.69%	72.09%	87.33%	89.4%	85.33%	94.63%	73.56%	88.35%
PZA ¹	25	138	0%	94.2%	0%	83.87%	79.75%	40%	71.74%	20.41%	86.84%	66.87%	Na	Na	Na	Na	Na	24%	61.59%	10.17%	81.73%	55.83%	68%	50.72%	20%	89.74%	53.37%
EMB ¹	67	213	82.09%	65.73%	42.97%	92.11%	69.64%	68.66%	75.12%	46.46%	88.4%	73.57%	64.18%	78.4%	48.31%	87.43%	75%	76.12%	67.61%	42.5%	90%	69.64%	80.59%	65.73%	42.52%	91.5%	69.28%
STR ²	39	75	56.41%	97.33%	91.67%	81.11%	83.33%	48.72%	88%	67.86%	76.74%	74.56%	43.59%	97.33%	89.47%	76.84%	78.95%	53.85%	88%	70%	78.57%	76.32%	69.23%	94.66%	87.09%	85.54%	85.96%
OFL ²	29	45	75.86%	91.11%	84.62%	85.42%	85.14%	55.17%	93.33%	84.21%	76.36%	78.38%	72.41%	88.89%	80.77%	83.33%	82.43%	86.21%	80%	73.53%	90%	82.43%	79.31%	82.22%	74.19%	86.04%	81.08%
AMK ²	12	68	75%	97.06%	81.82%	95.65%	93.75%	41.67%	98.53%	83.33%	90.54%	90%	75%	98.53%	90%	95.71%	95%	75%	91.18%	60%	95.38%	88.75%	75%	92.64%	64.28%	95.45%	90%
KAN ²	7	29	42.86%	100%	100%	87.88%	88.89%	28.57%	100%	100%	85.29%	86.11%	42.86%	96.55%	75%	87.5%	86.11%	57.14%	96.55%	80%	90.32%	88.89%	42.86%	96.55%	75%	87.5%	86.11%
CAP ²	7	29	42.86%	96.55%	75%	87.5%	86.11%	0%	80.56%	0%	100%	80.56%	42.86%	96.55%	75%	87.5%	86.11%	42.86%	96.55%	75%	87.5%	86.11%	42.86%	96.55%	75%	87.5%	86.11%

ATB = antibiotic; RES = resistant; SUS = susceptible; SEN = sensitivity; SPE = specificity; PPV = positive predictive value; NPV = negative predictive value; ACC = accuracy. ^1^ = first-line drugs; ^2^ = second-line drugs.

**Table 2:** Novel mutations observed in this study using TB Profiler 5.0.

n Isolates	Drug	DST	Novel Mutation	Phenotypic Profile	Genotypic Profile ‡
1	INH	R	*katG*_c.2070delC ^1^	MDR	p-XDR
1	INH	S	*katG*_c.1141dupG ^2^	RMR	IMR
1	ETH	NA	*ethA*_c.306_307delCA	MDR	p-XDR
1	ETH	NA	*ethA*_c.-382_*857del	p-XDR	p-XDR
1	ETH	NA	*ethA*_c.40dupA	MDR	MDR
4	ETH	NA	*ethA*_c.851dupC	MDR	2 MDR/2 p-XDR
4	PZA	2R/2NA	*pncA*_c.193_200dupTCCTCGTC	MDR	MDR
1	PZA	NA	*pncA*_c.289_293dupGGTGC	MDR	MDR
1	PZA	NA	*pncA*_c.502delA	Poly-R	Poly-R
1	PZA	R	*pncA*_c.452dupT	MDR	p-XDR
2	PZA	R	*pncA*_c.75_79delCGCGC	p-XDR	p-XDR
4	PZA	R/2S/NA	*pncA*_c.443_444dupGC ^3^	MDR	2 MDR/2 p-XDR
1	PZA	S	*pncA*_c.117_124delGGACTACC	MDR	p-XDR
1	PZA	S	*pncA*_c.300delC	MDR	Poly-R
2	PZA	R/NA	*pncA*_c.305dupC	p-XDR/Poly-R	p-XDR
1	PZA	S	*pncA*_c.329_338delACGAGAACGG	p-XDR	Poly-R
1	PZA	S	*pncA*_c.-3449_*7353del	p-XDR	p-XDR
1	PZA	S	*pncA*_c.423_424delGA	p-XDR	p-XDR
2	PZA	S	*pncA*_c.454_455insT	MDR	MDR
1	PZA	S	*pncA*_c.527dupG	MDR	MDR

‡ = genotypic resistance profile classified according to TB Profiler 5.0. ^1^ Genotypic isoniazid resistance profile = *ahpC*_c.-81C>T, *katG*_c.2070delC. ^2^ Genotypic isoniazid resistance profile = *ahpC*_c.-48G>A, *katG*_c.1141dupG. ^3^ Genotypic pyrazinamide resistance profile = *pncA*_c.443_444dupGC, *pncA*_p.Phe81Val. Isolate PZA phenotypically susceptible. * = deletion beyond the boundaries of coding sequence.

**Table 3:** Chronic TB patients with changes in genotypic profile mutations and phenotypic resistance.

			Resistance Mutation Genotypic Profile											Resistance Phenotypic Profile																				
Patient ID	Sample ID	Isolate Year	INH	RIF	EMB	FQ	STR	AMK	CAP	KAN	PZA	ETH	Gen-DR	RIF	INH	PZA	EMB	STR	AMI	OFL	KAN	CAP	Sub-Lineage	Homo SNPs	Het SNPs	Spoligofamily/SIT	Sex	Smoker	Diabetes	HIV	Lung Lesion	Lung Lesion	Clinical Outcome	SNP
																															(First Available)	(Last Available)		Dist
P28	G23103	2010	S315T	S450L	M306V		K43R	A1401G	A1401G	A1401G			MDR	R	R	R	R	R	R	S	R	R	L4.10/PGG3	833	39	T1/167								
P28	G23335	2011	S315T	S450L	M306V	S91P	K43R	A1401G	A1401G	A1401G			p-XDR	R	R	-	R	-	-	-	-	-	L4.10/PGG3	839	6	T1/167								
P28	G23216	2011	S315T	S450L	M306V	S91P	K43R	A1401G	A1401G	A1401G			p-XDR	R	R	-	R	-	-	-	-	-	L4.10/PGG3	834	51	T1/167	M	No	No	Yes	UC	UC	Death	0–1
																																		
P27	G23178	2008	S315T	S450L	M306V ^1^		K43R	G1484T	G1484T				MDR	R	R	R	R	R	-	-	-	-	L4.10/PGG3	751	62	T1/167								
P27	G23124	2009	S315T	S450L	M306V	A90V	K43R	G1484T	G1484T				p-XDR	R	R	S	S	-	R	R	-	-	L4.10/PGG3	837	20	T1/167	M	No	No	No	UC	BC	Death	0
																																		
P54	G23259	2012	S315T	S450L									MDR	R	R	S	S	-	-	-	-	-	L4.4/Vietnam	830	12	S/34								
P54	G23242	2012	S315T	S450L	G406D ^2^								MDR	R	R	S	S	-	-	-	-	-	L4.4/Vietnam	815	38	S/34	F	No	No	No	BC	BC	Death	0
																																		
P63	G23094	2010		D435V									RMR	R	R	-	S	R	-	-	-	-	L4.1.2/Harlem	800	33	ND/Orphan								
P63	G23327	2011		D435V		D94H							p-XDR	R	R	S	S	-	-	-	-	-	L4.1.2/Harlem	805	10	ND/Orphan	M	No	Yes	No	BC	BC	Cured	3
																																		
P35	G23277	2012	S315T ^2^	Q432P									MDR	R	R	S	S	-	-	-	-	-	L4.1.1/X	608	177	T1/53								
P35	G23280	2012	S315T	S450L	Q497R								MDR	R	R	S	S	-	-	-	-	-	L4.3/LAM	829	44	ND/Orphan	F	No	No	No	BC	BC	Abandonment	484
																																		
P10	G23108	2008	S315T	S450L	M306V								MDR	R	R	-	S	S	-	-	-	-	L4.3/LAM	833	20	LAM6/64								
P10	G23167	2012	S315T	S450L	M306V	D94A							p-XDR	R	R	-	S	-	-	-	-	-	L4.3/LAM	826	52	LAM6/64	M	No	No	Yes	BC	BC	Death	2
																																		
P39	G23185	2008	C-15T/S94A	S450L	G406C							C-15T/S94A	MDR	S	R	R	-	-	S	-	-	-	L4.3/LAM	849	25	LAM6/Orphan								
P39	G23090	2010	C-15T/S94A	S450L	G406C	D94G/A90V						C-15T/S94A	p-XDR	R	R	-	R	S	R	R	S	S	L4.3/LAM	837	18	LAM6/Orphan	F	No	Yes	No	BC	BC	Death	1

^1^ In addition to the mutation M306V, there was a gap in codons 405 and 406, which resulted in flag G→T E405D, G→C E405D, G→T G406C, G→A G406S, G→A G406D, and G→C G406A. ^2^ Mixed SNP call. ND = not determined. Homo SNPs = all bases detected in the same genome position are the same. Het SNP = bases detected in the same genome position are different. UC = unilateral cavitary. BC = bilateral cavitary.

## Data Availability

Data are contained within the article.

## Supplementary Materials

The following supporting information can be downloaded at https://www.mdpi.com/article/10.3390/antibiotics13060496/s1. Figure S1: Lineage classification of 298 genome isolates using four pipelines. Figure S2: Venn diagram for the four pipelines used in lineage classification of the 298 genome isolates. Table S1: Phenotypic profile of 294 isolates included in the study. Table S2: Clinical data of 112 patients obtained from SITETB and SINAN databases. Table S3: Resistance-related mutations detected by the in-house WGS pipeline in 298 isolates. Table S4: Agreement among phenotypic DST, in-house WGS pipeline, KvarQ, Mykrobe Predictor, and TB Profiler versions 0.3.4 and 5.0 according to free marginal kappa coefficient. Table S5: Resistance-related mutations detected by TB Profiler version 5.0 in 298 isolates. Table S6: Chronic patients without changes in genotypic profile mutation resistance. Table S7: Genotypic and phenotypic profiles of the 82 patients with cured and deceased treatment outcomes.
