# Supplementary material for: Genetic Characterization and Population Structure of Drug-Resistant Mycobacterium tuberculosis Isolated from Brazilian Patients Using Whole-Genome Sequencing
Source: Antibiotics (Basel). 2024 May 28;13(6):496. doi: 10.3390/antibiotics13060496 (PMC11200758; doi:10.3390/antibiotics13060496)
Supplement: Supplementary file 1 [file antibiotics-13-00496-s001.zip › Supplementary Figures 10.04.24.docx]

**Supplementary Figure S1.** Lineage classification of 298 genomes isolates using four pipelines.
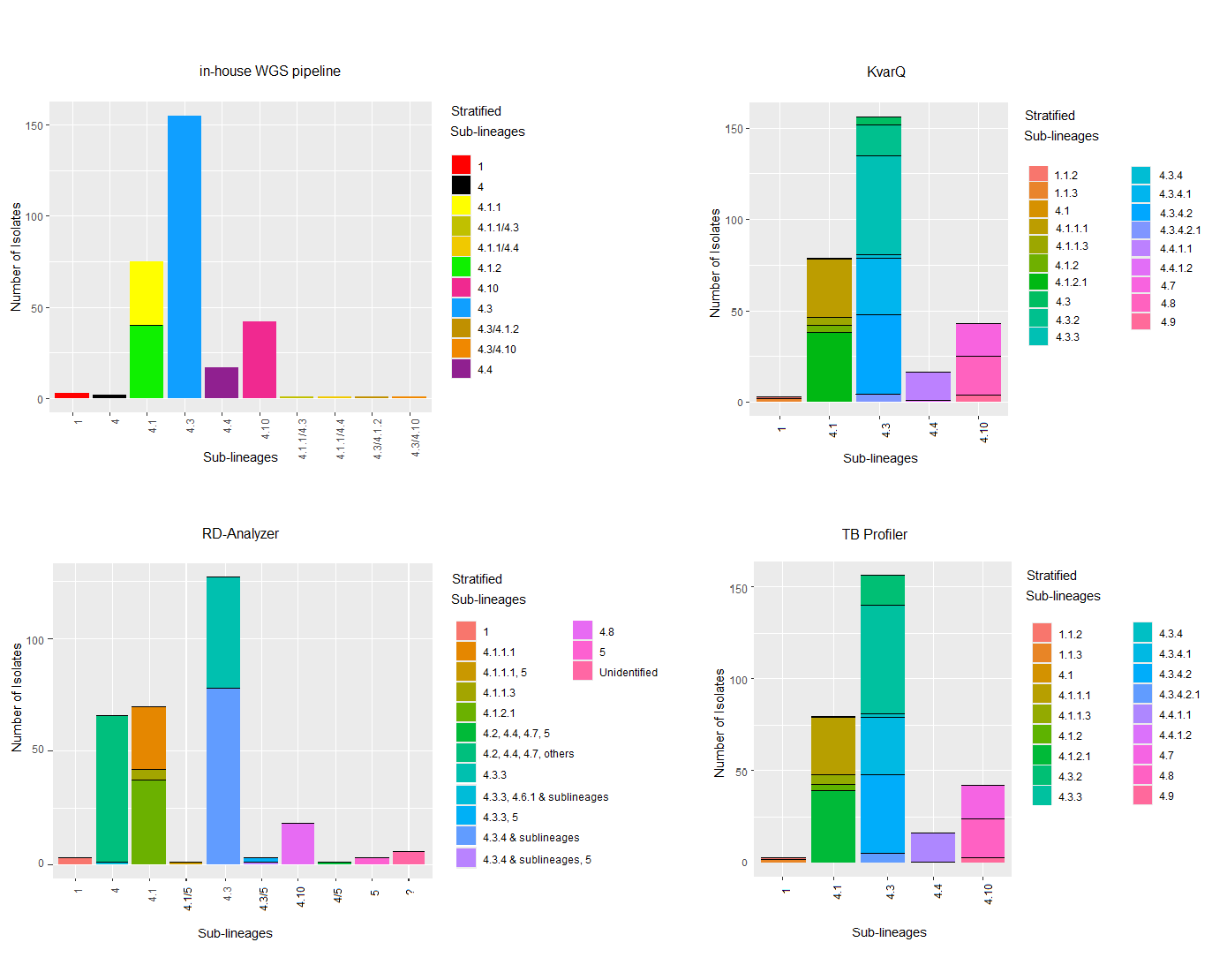


Three SNP-based pipelines (*in-house* WGS pipeline, kvarQ-barcode-Coll14 and TB Profiler) and a RD-based analyze pipeline (RD-Analyzer) were used.

**Supplementary Figure S2.** Venn diagram for the four pipelines used in lineage classification of the 298 genomes isolates.


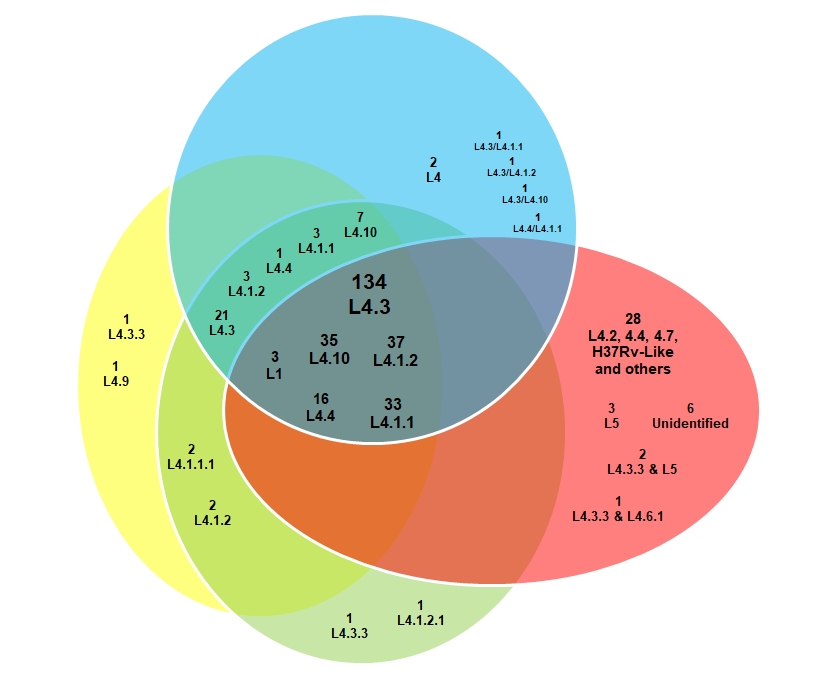


Blue: *in-house* WGS pipeline; Yellow: KvarQ-barcode-Coll14; Green: TB Profiler; and Red: RD-Analyzer. The Differences only in resolution classifications were considered as concordant. L4.10 is a comprehensive sub-lineage comprising sub-lineages L4.7, L4.8 and L4.9.
