# Supplementary material for: Genetic Characterization and Population Structure of Drug-Resistant Mycobacterium tuberculosis Isolated from Brazilian Patients Using Whole-Genome Sequencing
Source: Antibiotics (Basel). 2024 May 28;13(6):496. doi: 10.3390/antibiotics13060496 (PMC11200758; doi:10.3390/antibiotics13060496)
Supplement: Supplementary file 1 [file antibiotics-13-00496-s001.zip › Supplementary Tables 10.04.24.docx]

**Table S1.** Phenotypic profile of 294 isolates included in the study.

|  | |
| --- | --- |
| ***n* Isolates (%)** | **Phenotypic profile** |
| **161 (54.76%)** | **MDR** |
| 23 | MDR (STR, AMK, OFL, KAN, CAP DST NA) |
| 20 | MDR (PZA, STR, AMK, OFL, KAN, CAP DST NA) |
| 19 | MDR + EMB-R (STR, AMK, OFL, KAN, CAP DST NA) |
| 11 | MDR + EMB-R (PZA, STR, AMK, OFL, KAN, CAP DST NA) |
| 10 | MDR (AMK, OFL, KAN, CAP DST NA) |
| 7 | MDR (full DST available) |
| 7 | MDR (PZA, AMK, OFL, KAN, CAP DST NA) |
| 6 | MDR + PZA-R (STR, AMK, OFL, KAN, CAP DST NA) |
| 5 | MDR + PZA-R (AMK, OFL, KAN, CAP DST NA) |
| 5 | MDR + STR-R (PZA DST NA) |
| 4 | MDR (KAN, CAP DST NA) |
| 4 | MDR + EMB-R (AMK, OFL, KAN, CAP DST NA) |
| 3 | MDR (PZA DST NA) |
| 3 | MDR + STR-R (KAN, CAP DST NA) |
| 3 | MDR + STR-R (AMK, OFL, KAN, CAP DST NA) |
| 3 | MDR + STR-R (PZA, AMK, OFL, KAN, CAP DST NA) |
| 2 | MDR + EMB-R (PZA DST NA) |
| 2 | MDR + EMB-R (KAN, CAP DST NA) |
| 2 | MDR + EMB-R (PZA, AMK, OFL, KAN, CAP DST NA) |
| 2 | MDR + EMB-R, STR-R (PZA, AMK, OFL, KAN, CAP DST NA) |
| 1 | MDR + STR-R (full DST available) |
| 1 | MDR (STR DST NA) |
| 1 | MDR + EMB-R (full DST available) |
| 1 | MDR + EMB-R + SRT-R (full DST available) |
| 1 | MDR + PZA-R + STR-R (EMB DST NA) |
| 1 | MDR (STR, KAN, CAP DST NA) |
| 1 | MDR + PZA-R (KAN, CAP DST NA) |
| 1 | MDR (PZA, KAN, CAP DST NA) |
| 1 | MDR + EMB-R + STR-R (KAN, CAP DST NA) |
| 1 | MDR + STR-R (EMB, KAN, CAP DST NA) |
| 1 | MDR (EMB, STR, OFL, KAN, CAP DST NA) |
| 1 | MDR + PZA-R + STR-R (AMK, OFL, KAN, CAP DST NA) |
| 1 | MDR + EMB-R + STR-R (AMK, OFL, KAN, CAP DST NA) |
| 1 | MDR + PZA-R, EMB-R, STR-R (AMK, OFL, KAN, CAP DST NA) |
| 1 | MDR + STR-R (PZA, EMB, AMK, OFL, KAN, CAP DST NA) |
| 1 | MDR + EMB-R, AMK-R (STR, KAN, CAP DST NA) |
| **27 (9.18%)** | **p-XDR** |
| 4 | MDR + OFL-R (KAN, CAP DST NA) |
| 3 | MDR + OFL-R + EMB-R (KAN, CAP DST NA) |
| 2 | MDR + OFL-R (PZA DST NA) |
| 1 | MDR + OFL-R (full DST available) |
| 1 | MDR + OFL-R +STR-R (full DST available) |
| 1 | MDR + OFL-R (STR DST NA) |
| 1 | MDR + OFL-R + STR-R (KAN, CAP DST NA) |
| 1 | MDR + OFL-R (STR, KAN, CAP DST NA) |
| 1 | MDR + OFL-R + EMB-R + STR-R (KAN, CAP DST NA) |
| 1 | MDR + OFL-R + EMB-R + PZA-R (STR, KAN, CAP DST NA) |
| 1 | RIF-R + OFL-R (full DST available) |
| 1 | MDR + OFL-R + STR-R + PZA-R (EMB, KAN, CAP DST NA) |
| 2 | MDR + OFL-R + EMB-R + STR-R + AMK-R + CAP-R + KAN-R (full DST available) |
| 1 | MDR + OFL-R + AMK-R + EMB-R (PZA DST NA) |
| 1 | MDR + OFL-R + PZA-R + STR-R + AMK-R + CAP-R + KAN-R (full DST available) |
| **10 (3.4%)** | **Poly-R** |
| 2 | INH-R + EMB-R (PZA, STR, AMK, OFL, KAN, CAP DST NA) |
| 1 | INH-R + EMB-R (KAN, CAP DST NA) |
| 1 | INH-R + EMB-R + OFL-R (PZA, KAN, CAP DST NA) |
| 1 | INH-R + AMK-R + OFL-R (STR, KAN, CAP DST NA) |
| 1 | INH-R + PZA-R + STR-R (OFL, KAN, CAP DST NA) |
| 1 | INH-R + PZA-R + STR-R (EMB, OFL, KAN, CAP DST NA) |
| 1 | INH-R + PZA-R (EMB, STR, OFL, KAN, CAP DST NA) |
| 1 | RIF-R + EMB-R (full DST available) |
| 1 | RIF-R + PZA-R (STR, AMK, OFL, KAN, CAP DST NA) |
| 2 | INH-R + EMB-R (PZA, STR, AMK, OFL, KAN, CAP DST NA) |
| **22 (7.5%)** | **IMR** |
| 15 | IMR (PZA, STR, AMK, OFL, KAN, CAP DST NA) |
| 3 | IMR (STR, AMK, OFL, KAN, CAP DST NA) |
| 2 | IMR (EMB, AMK, OFL, KAN, CAP DST NA) |
| 1 | IMR (STR, KAN, CAP DST NA) |
| 1 | IMR (EMB, STR, AMK, OFL, KAN, CAP DST NA) |
| **9 (3.07%)** | **RMR** |
| 5 | RMR (PZA, STR, AMK, OFL, KAN, CAP DST NA) |
| 2 | RMR (AMK, OFL, KAN, CAP DST NA) |
| 1 | RMR (STR, OFL, KAN, CAP DST NA) |
| 1 | RMR (PZA, AMK, OFL, KAN, CAP DST NA) |
| **1 (0.34%)** | **EMR** |
| 1 | EMR (STR, AMK, OFL, KAN, CAP DST NA) |
| **64 (21.84%)** | **Pan-susceptible** |
| 44 | Pan-Susceptible (PZA, STR, AMK, OFL, KAN, CAP DST NA) |
| 11 | Pan-Susceptible (STR, AMK, OFL, KAN, CAP DST NA) |
| 2 | Pan-Susceptible (AMK, OFL, KAN, CAP DST NA) |
| 1 | Pan-Susceptible (KAN, CAP DST NA) |
| 1 | Pan-Susceptible (STR, KAN, CAP DST NA) |
| 1 | Pan-Susceptible (EMB, KAN, CAP DST NA) |
| 1 | Pan-Susceptible (EMB, AMK, OFL, KAN, CAP DST NA) |
| 1 | Pan-Susceptible (PZA, EMB, STR, AMK, OFL, KAN, CAP DST NA) |
| 1 | Pan-Susceptible (INH, AMK, OFL, KAN, CAP DST NA) |
| 1 | Pan-Susceptible (INH, PZA, STR, AMK, OFL, KAN, CAP DST NA) |
|  |  |

MDR = Multidrug-resistant, p-XDR = Pre-Extensively drug-resistant, Poly-R = Poly drug-resistant, IMR = Isoniazid monoresistant, RMR = Rifampicin monoresistant, EMR = Ethambutol monoresistant, NA = not available.

**Table S2.** Clinical data of 112 patients obtained from the SITETB and SINAN databases.

| ***n* Patients** | **Outcome** | **HIV** | **Smoker** | **Illicit drugs** | **Diabetes** | **Alcoholic addict** | **Lung lesion** |
| --- | --- | --- | --- | --- | --- | --- | --- |
| 1 | NA | No | No | No | No | No | NA |
| 1 | NA | No | No | No | Yes | No | Bilateral Cavitary |
| 1 | NA | No | No | No | No | No | Bilateral non-Cavitary |
| 1 | NA | No | No | No | No | No | Unilateral Cavitary |
| 2 | Abandonment | No | No | No | No | No | NA |
| 1 | Abandonment | NA | Yes | No | No | Yes | Bilateral Cavitary |
| 8 | Abandonment | No | No | No | No | No | Bilateral Cavitary |
| 1 | Abandonment | No | Yes | No | No | No | Bilateral Cavitary |
| 1 | Abandonment | No | No | Yes | No | No | Bilateral Cavitary |
| 1 | Abandonment | No | No | No | Yes | No | Bilateral Cavitary |
| 2 | Abandonment | No | No | No | No | No | Unilateral Cavitary |
| 2 | Cured | NA | NA | NA | NA | NA | NA |
| 1 | Cured | NA | No | No | No | No | NA |
| 1 | Cured | No | NA | NA | NA | NA | NA |
| 3 | Cured | No | No | No | No | No | NA |
| 2 | Cured | NA | No | No | No | No | Bilateral Cavitary |
| 22 | Cured | No | No | No | No | No | Bilateral Cavitary |
| 1 | Cured | No | Yes | No | No | No | Bilateral Cavitary |
| 4 | Cured | No | No | No | Yes | No | Bilateral Cavitary |
| 1 | Cured | No | No | No | No | Yes | Bilateral Cavitary |
| 1 | Cured | Yes | No | No | No | No | Bilateral Cavitary |
| 3 | Cured | No | No | No | No | No | Bilateral non-Cavitary |
| 1 | Cured | No | No | No | Yes | No | Bilateral non-Cavitary |
| 1 | Cured | NA | Yes | No | No | Yes | Unilateral Cavitary |
| 6 | Cured | No | No | No | No | No | Unilateral Cavitary |
| 1 | Cured | No | No | No | Yes | No | Unilateral non-Cavitary |
| 4 | Deceased | No | No | No | No | No | NA |
| 1 | Deceased | No | No | Yes | No | Yes | NA |
| 1 | Deceased | Yes | No | Yes | No | Yes | NA |
| 1 | Deceased | NA | No | No | No | No | Bilateral Cavitary |
| 9 | Deceased | No | No | No | No | No | Bilateral Cavitary |
| 1 | Deceased | No | No | Yes | No | No | Bilateral Cavitary |
| 3 | Deceased | No | No | No | Yes | No | Bilateral Cavitary |
| 2 | Deceased | No | No | No | No | Yes | Bilateral Cavitary |
| 1 | Deceased | No | Yes | No | No | Yes | Bilateral Cavitary |
| 1 | Deceased | No | No | Yes | No | Yes | Bilateral Cavitary |
| 1 | Deceased | No | No | Yes | Yes | Yes | Bilateral Cavitary |
| 1 | Deceased | Yes | No | No | No | No | Bilateral Cavitary |
| 1 | Deceased | Yes | No | No | No | No | Bilateral non-Cavitary |
| 3 | Deceased | No | No | No | No | No | Unilateral Cavitary |
| 1 | Deceased | Yes | No | No | No | No | Unilateral Cavitary |
| 1 | Deceased | Yes | Yes | No | No | No | Unilateral non-Cavitary |
| 1 | Deceased by another cause | No | No | No | No | No | Bilateral Cavitary |
| 2 | Deceased by another cause | No | No | No | Yes | No | Bilateral Cavitary |
| 1 | Deceased by another cause | No | No | No | Yes | No | Unilateral Cavitary |
| 1 | Deceased by another cause | Yes | No | Yes | No | No | Unilateral non-Cavitary |
| 2 | Under Treatment | No | Yes | No | No | No | Bilateral Cavitary |
| 1 | Under Treatment | No | No | No | Yes | No | Bilateral Cavitary |
| 1 | Under Treatment | No | No | No | No | Yes | Bilateral Cavitary |
| 1 | Under Treatment | No | No | Yes | No | Yes | Bilateral Cavitary |
| 1 | NA | No | No | No | No | No | NA |

**NA = not available.**

**Table S3.** Mutations resistance related detected by the in-house WGS pipeline in 298 isolates.

| ***n* Isolates** | **Gene** | **Base Mutation** | **Amino acid Mutation** | **Drug  Resistance** |
| --- | --- | --- | --- | --- |
| 172 | katG | C→G | S315T | INH |
| 6 | katG | G→C | S315R | INH |
| 3 | inhA | T→C | I194T | INH |
| 3 | katG | C→A | S315I | INH |
| 2 | katG | G→C | P232R | INH |
| 2 | katG | C→T | S315N | INH |
| 1 | katG | T→G | S315R | INH |
| 1 | katG | G→T | S315R | INH |
| 28 | fabG1 | C→T | C-15T | INH;ETH |
| 5 | inhA | T→G | S94A | INH;ETH |
| 2 | fabG1 | T→C | T-8C | INH;ETH |
| 1 | fabG1 | G→T | G-17T | INH;ETH |
| 136 | rpoB | C→T | S450L | RIF |
| 13 | rpoB | G→T | D435Y | RIF |
| 11 | rpoB | A→T | D435V | RIF |
| 7 | rpoB | C→T | H445Y | RIF |
| 6 | rpoB | C→G | H445D | RIF |
| 6 | rpoB | A→G | H445R | RIF |
| 6 | rpoB | A→C | Q432P | RIF |
| 3 | rpoB | C→A | H445N | RIF |
| 3 | rpoB | C→G | S450W | RIF |
| 2 | rpoB | T→C | L452P | RIF |
| 2 | rpoB | C→T | S441L | RIF |
| 1 | rpoB | A→T | H445L | RIF |
| 1 | rpoB | A→T | I491F | RIF |
| 51 | embB | A→G | M306V | EMB |
| 21 | embB | G→A | M306I | EMB |
| 15 | embB | G→C | G406A | EMB |
| 14 | embB | A→G | Q497R | EMB |
| 11 | embB | G→A | G406D | EMB |
| 10 | embB | G→A | G406S | EMB |
| 10 | embB | C→A | Q497K | EMB |
| 9 | embA | C→T | C-12T | EMB |
| 9 | embB | G→T | G406C | EMB |
| 8 | embB | G→C | M306I | EMB |
| 6 | embB | G→C | E405D | EMB |
| 6 | embB | G→T | M306I | EMB |
| 5 | embB | G→T | E405D | EMB |
| 4 | embB | A→C | D354A | EMB |
| 2 | embB | G→A | D1024N | EMB |
| 1 | embB | C→T | A505V | EMB |
| 1 | embB | A→C | M306L | EMB |
| 6 | pncA | T→C | A-11G | PZA |
| 3 | pncA | G→A | Q10STOP | PZA |
| 2 | pncA | G→A | Q141STOP | PZA |
| 2 | pncA | T→C | T-12C | PZA |
| 1 | pncA | A→G | C14R | PZA |
| 1 | pncA | T→G | D12A | PZA |
| 1 | pncA | C→T | D49N | PZA |
| 1 | pncA | T→C | D8G | PZA |
| 1 | pncA | C→T | D8N | PZA |
| 1 | pncA | C→A | G78C | PZA |
| 1 | pncA | G→C | H57D | PZA |
| 1 | pncA | T→C | H57R | PZA |
| 1 | pncA | A→G | L27P | PZA |
| 1 | pncA | A→G | L4S | PZA |
| 1 | pncA | G→T | S104R | PZA |
| 1 | pncA | C→G | V7L | PZA |
| 1 | pncA | C→A | W68C | PZA |
| 1 | pncA | C→G | W68C | PZA |
| 21 | gyrA | C→T | A90V | FQ |
| 18 | gyrA | A→G | D94G | FQ |
| 11 | gyrA | T→C | S91P | FQ |
| 6 | gyrA | A→C | D94A | FQ |
| 6 | gyrA | G→T | D94Y | FQ |
| 4 | gyrA | G→C | D94H | FQ |
| 4 | gyrA | G→A | D94N | FQ |
| 1 | gyrA | G→C | G88A | FQ |
| 14 | rrs | A→G | A1401G | AMK;CAP;KAN |
| 4 | rrs | G→T | G1484T | AMK;CAP |
| 4 | eis | G→A | C-14T | KAN;CAP |
| 1 | eis | C→T | G-10A | KAN |
| 4 | ethA | G→A | P192S | ETH |
| 3 | ethA | C→T | A175T | ETH |
| 3 | ethA | A→G | C137R | ETH |
| 3 | ethA | T→G | H281P | ETH |
| 3 | ethA | T→C | I337V | ETH |
| 3 | ethA | A→C | L136R | ETH |
| 3 | ethA | G→T | N345K | ETH |
| 3 | ethA | G→C | N345K | ETH |
| 3 | ethA | G→A | P230L | ETH |
| 3 | ethA | G→C | P334A | ETH |
| 3 | ethA | T→G | Q165P | ETH |
| 3 | ethA | T→C | Q246R | ETH |
| 3 | ethA | G→T | S266R | ETH |
| 3 | ethA | G→C | S266R | ETH |
| 3 | ethA | T→G | S266R | ETH |
| 3 | ethA | C→G | W167S | ETH |
| 3 | ethA | C→T | W167STOP | ETH |
| 3 | ethA | C→T | W167STOP | ETH |
| 3 | ethA | C→T | W256STOP | ETH |
| 3 | ethA | C→T | W256STOP | ETH |
| 3 | ethA | A→C | Y32D | ETH |
| 23 | rpsL | A→G | K43R | SM |
| 12 | rpsL | A→G | K88R | SM |
| 5 | rrs | C→T | C517T | SM |
| 4 | gid | A→C | V65G | SM |
| 2 | rrs | A→G | A906G | SM |
| 1 | rrs | A→C | A514C | SM |
| 1 | gid | A→G | L79S | SM |

**Table S4.** Agreement among DST phenotypic test, in-house WGS pipeline, KvarQ, Mykrobe Predictor, TB Profiler version 0.3.4 and 5.0 according free-marginal kappa coefficient.

| **Drug** | ***n* samples** | **% Overall  agreement** | **Free-marginal  kappa** | **95% CI** | **Agreement level** |
| --- | --- | --- | --- | --- | --- |
| RIF | 294 | 94.5% | 0.89 | 0.86 - 0.92 | Excellent |
| INH | 292 | 93.1% | 0.86 | 0.83 - 0.90 | Excellent |
| PZA* | 163 | 65.7% | 0.32 | 0.23 - 0.40 | Poor |
| EMB | 280 | 84.2% | 0.69 | 0.63 - 0.74 | Moderate-Good |
| STR | 114 | 85.7% | 0.71 | 0.63 - 0.79 | Moderate-Good |
| OFX | 74 | 86.2% | 0.72 | 0.63 - 0.82 | Moderate-Good |
| AMK | 80 | 93.2% | 0.87 | 0.80 - 0.93 | Excellent |
| KAN | 36 | 93.1% | 0.86 | 0.76 - 0.97 | Excellent |
| CAP | 36 | 92.0% | 0.84 | 0.74 - 0.94 | Excellent |

*PZA resistance prediction was not available for Mykrobe Predictor.

**Table S5.** Mutations resistance-related detected by TB Profiler version 5.0 in 298 isolates.

| **Gene/Mutation** | **Drug** | **Novel mutation** | **Mutation not present in WHO  catalogue** |
| --- | --- | --- | --- |
| rpoB_p.Ala286Val | RIF |  |  |
| rpoB_p.Asp435Phe | RIF |  |  |
| rpoB_p.Asp435Tyr | RIF |  |  |
| rpoB_p.Asp435Val | RIF |  |  |
| rpoB_p.Gln432Pro | RIF |  |  |
| rpoB_p.His445Arg | RIF |  |  |
| rpoB_p.His445Asn | RIF |  |  |
| rpoB_p.His445Asp | RIF |  |  |
| rpoB_p.His445Cys | RIF |  |  |
| rpoB_p.His445Leu | RIF |  |  |
| rpoB_p.His445Ser | RIF |  |  |
| rpoB_p.His445Tyr | RIF |  |  |
| rpoB_p.Ile491Phe | RIF |  |  |
| rpoB_p.Leu430Pro | RIF |  |  |
| rpoB_p.Leu449Met | RIF |  |  |
| rpoB_p.Leu452Pro | RIF |  |  |
| rpoB_p.Met434Ile | RIF |  |  |
| rpoB_p.Phe424Val | RIF |  |  |
| rpoB_p.Phe433Val¹ | RIF |  | X |
| rpoB_p.Pro454Ser | RIF |  |  |
| rpoB_p.Ser428Arg | RIF |  |  |
| rpoB_p.Ser428Thr | RIF |  |  |
| rpoB_p.Ser441Leu | RIF |  |  |
| rpoB_p.Ser450Leu | RIF |  |  |
| rpoB_p.Ser450Phe | RIF |  |  |
| rpoB_p.Ser450Trp | RIF |  |  |
| rpoB_p.Ser450Tyr | RIF |  |  |
| rpoB_p.Ser493Leu | RIF |  |  |
| rpoC_p.Gly332Arg | RIF |  |  |
| rpoC_p.Leu527Val | RIF |  |  |
| ahpC_c.-48G>A | INH |  |  |
| ahpC_c.-54C>T | INH |  |  |
| ahpC_c.-81C>T | INH |  |  |
| fabG1_c.-15C>T | INH and ETH |  |  |
| fabG1_c.-17G>T | INH and ETH |  |  |
| fabG1_c.-8T>C | INH and ETH |  |  |
| inhA_c.-154G>A | INH and ETH |  |  |
| inhA_p.Ile194Thr | INH and ETH |  |  |
| inhA_p.Ser94Ala | INH and ETH |  |  |
| katG_c.1141dupG | INH | X | X |
| katG_c.1284delG | INH |  |  |
| katG_c.2070delC | INH | X | X |
| katG_p.Ala379Val | INH |  |  |
| katG_p.Asn138Ser | INH |  |  |
| katG_p.Leu634Phe | INH |  | X |
| katG_p.Met257Ile | INH |  |  |
| katG_p.Ser140Asn | INH |  |  |
| katG_p.Ser315Arg | INH |  |  |
| katG_p.Ser315Asn | INH |  |  |
| katG_p.Ser315Ile | INH |  |  |
| katG_p.Ser315Thr | INH |  |  |
| embA_c.-11C>A | EMB |  |  |
| embA_c.-12C>T | EMB |  |  |
| embA_c.-16C>T | EMB |  |  |
| embB_p.Asp1024Asn | EMB |  |  |
| embB_p.Asp354Ala | EMB |  |  |
| embB_p.Gln497Arg | EMB |  |  |
| embB_p.Gln497Lys | EMB |  |  |
| embB_p.Glu405Asp | EMB |  |  |
| embB_p.Glu504Asp | EMB |  |  |
| embB_p.Gly406Ala | EMB |  |  |
| embB_p.Gly406Asp | EMB |  |  |
| embB_p.Gly406Cys | EMB |  |  |
| embB_p.Gly406Ser | EMB |  |  |
| embB_p.Met306Ile | EMB |  |  |
| embB_p.Met306Leu | EMB |  |  |
| embB_p.Met306Val | EMB |  |  |
| pncA_c.117_124delGGACTACC | PZA | X | X |
| pncA_c.-11A>G | PZA |  |  |
| pncA_c.-12T>G | PZA |  | X |
| pncA_c.193_200dupTCCTCGTC | PZA | X | X |
| pncA_c.289_293dupGGTGC | PZA | X | X |
| pncA_c.300delC | PZA | X | X |
| pncA_c.305dupC | PZA | X | X |
| pncA_c.329_338delACGAGAACGG | PZA | X | X |
| pncA_c.-3449_*7353del | PZA | X | X |
| pncA_c.423_424delGA | PZA | X | X |
| pncA_c.443_444dupGC | PZA | X | X |
| pncA_c.452dupT | PZA | X | X |
| pncA_c.454_455insT | PZA | X | X |
| pncA_c.502delA | PZA | X | X |
| pncA_c.521_522insT | PZA |  | X |
| pncA_c.527dupG | PZA | X | X |
| pncA_c.75_79delCGCGC | PZA | X | X |
| pncA_p.Ala161Pro | PZA |  |  |
| pncA_p.Ala171Glu | PZA |  |  |
| pncA_p.Ala171Thr | PZA |  |  |
| pncA_p.Asp12Asn | PZA |  |  |
| pncA_p.Asp49Ala | PZA |  |  |
| pncA_p.Asp49Glu | PZA |  |  |
| pncA_p.Asp63Ala | PZA |  |  |
| pncA_p.Asp63His | PZA |  | X |
| pncA_p.Cys138Tyr | PZA |  | X |
| pncA_p.Cys72Arg | PZA |  |  |
| pncA_p.Gln10* | PZA |  |  |
| pncA_p.Gln141* | PZA |  |  |
| pncA_p.Gln141Pro | PZA |  |  |
| pncA_p.Glu15* | PZA |  |  |
| pncA_p.Gly23Val | PZA |  | X |
| pncA_p.His137Pro | PZA |  |  |
| pncA_p.His51Pro | PZA |  |  |
| pncA_p.His57Tyr | PZA |  |  |
| pncA_p.Leu151Ser | PZA |  |  |
| pncA_p.Leu159Pro | PZA |  |  |
| pncA_p.Leu159Val | PZA |  |  |
| pncA_p.Leu85Pro | PZA |  |  |
| pncA_p.Lys96Asn | PZA |  |  |
| pncA_p.Met175Ile | PZA |  |  |
| pncA_p.Phe81Val | PZA |  |  |
| pncA_p.Pro54Leu | PZA |  |  |
| pncA_p.Pro62Leu | PZA |  |  |
| pncA_p.Pro62Ser | PZA |  |  |
| pncA_p.Ser104Arg | PZA |  |  |
| pncA_p.Ser164Pro | PZA |  |  |
| pncA_p.Thr142Met | PZA |  |  |
| pncA_p.Thr76Ile | PZA |  |  |
| pncA_p.Trp119Cys | PZA |  |  |
| pncA_p.Trp68Gly | PZA |  |  |
| pncA_p.Trp68Leu | PZA |  |  |
| pncA_p.Tyr103* | PZA |  |  |
| pncA_p.Tyr103His | PZA |  |  |
| pncA_p.Tyr34* | PZA |  |  |
| pncA_p.Tyr95* | PZA |  |  |
| pncA_p.Tyr99* | PZA |  |  |
| pncA_p.Val128Gly | PZA |  |  |
| pncA_p.Val131Phe | PZA |  |  |
| pncA_p.Val139Ala | PZA |  |  |
| pncA_p.Val139Gly | PZA |  |  |
| pncA_p.Val155Ala | PZA |  |  |
| pncA_p.Val155Gly | PZA |  |  |
| pncA_p.Val7Gly | PZA |  |  |
| gyrA_p.Ala90Val | FQ |  |  |
| gyrA_p.Asp89Asn | FQ |  |  |
| gyrA_p.Asp94Ala | FQ |  |  |
| gyrA_p.Asp94Asn | FQ |  |  |
| gyrA_p.Asp94Gly | FQ |  |  |
| gyrA_p.Asp94His | FQ |  |  |
| gyrA_p.Asp94Tyr | FQ |  |  |
| gyrA_p.Gly88Ala | FQ |  |  |
| gyrA_p.His70Arg | FQ |  |  |
| gyrA_p.Ser91Pro | FQ |  |  |
| gyrB_p.Ala504Val | FQ |  |  |
| gyrB_p.Asn499Thr | FQ |  |  |
| gyrB_p.Asp461Asn | FQ |  |  |
| gyrB_p.Asp461His | FQ |  |  |
| gyrB_p.Glu501Asp | FQ |  |  |
| gyrB_p.Glu501Val | FQ |  |  |
| gid_c.102delG | STR |  |  |
| gid_c.115delC | STR |  |  |
| gid_c.351delG | STR |  |  |
| gid_c.351dupG | STR |  |  |
| gid_p.Arg102* | STR |  |  |
| gid_p.Gln125* | STR |  |  |
| gid_p.Gln127* | STR |  |  |
| gid_p.Glu92* | STR |  |  |
| gid_p.Glu99* | STR |  |  |
| gid_p.Gly28* | STR |  |  |
| gid_p.Leu79Ser | STR |  |  |
| gid_p.Tyr195* | STR |  |  |
| rpsL_p.Lys43Arg | STR |  |  |
| rpsL_p.Lys88Arg | STR |  |  |
| rpsL_p.Lys88Gln | STR |  |  |
| rpsL_p.Lys88Thr | STR |  |  |
| rrs_n.514A>C | STR |  |  |
| rrs_n.517C>T | STR |  |  |
| rrs_n.878G>A | STR |  |  |
| rrs_n.906A>G | STR |  |  |
| eis_c.-14C>T | KAN;CAP |  |  |
| rrs_n.1401A>G | AMK;CAP;KAN |  |  |
| rrs_n.1484G>T | AMK;CAP |  |  |
| eis_c.-10G>A | KAN |  |  |
| ethA_c.1054delG | ETH |  |  |
| ethA_c.110delA | ETH |  |  |
| ethA_c.1290delC | ETH |  | X |
| ethA_c.140delT | ETH |  |  |
| ethA_c.306_307delCA | ETH | X | X |
| ethA_c.341delA | ETH |  |  |
| ethA_c.-382_*857del | ETH | X | X |
| ethA_c.40dupA | ETH | X | X |
| ethA_c.672_673dupGC | ETH |  |  |
| ethA_c.672dupG | ETH |  | X |
| ethA_c.752dupG | ETH |  |  |
| ethA_c.851dupC | ETH | X | X |
| ethA_p.Ala341Val | ETH |  |  |
| ethA_p.Arg463Ser | ETH |  | X |
| ethA_p.Cys131* | ETH |  |  |
| ethA_p.Gln254* | ETH |  |  |
| ethA_p.Glu132* | ETH |  |  |
| ethA_p.His22Pro | ETH |  |  |
| ethA_p.Leu397Arg | ETH |  |  |
| ethA_p.Trp391* | ETH |  |  |
| ethA_p.Tyr84Asp | ETH |  |  |
| ethR_p.Phe110Leu | ETH |  |  |
| folC_p.Glu40Gly | PAS |  |  |
| folC_p.Ile43Thr | PAS |  |  |
| folC_p.Ser150Gly | PAS |  |  |
| thyX_c.-16C>T | PAS |  |  |
| ald_c.1107dupG | CSE |  |  |
| ald_c.130dupG | CSE |  |  |
| ald_c.436_437dupGC | CSE |  |  |
| ald_c.464delG | CSE |  |  |
| alr_p.Met343Thr | CSE |  |  |

PAS = Para aminosalicylic Acid; CSE = Cycloserine
¹ Genotypic rifampicin resistance profile = rpoB_p.Gln432Pro, rpoB_p.Phe433Val, rpoB_p.Ser450Leu

Table S6. Chronical patients without changes in genotypic profile mutation resistance

|  |  | |  | |  | | | | | | | | | | | | | | | | | | | |  | |  | | | | | | | | | | | | | | | | | |  | |  | |  | | |  | |  | |  | |  | |  | |  | | | |  | | | |  |  | | | | |  | | | |  | |  | | | |  | |  |  |
| --- | --- | --- | --- | --- | --- | --- | --- | --- | --- | --- | --- | --- | --- | --- | --- | --- | --- | --- | --- | --- | --- | --- | --- | --- | --- | --- | --- | --- | --- | --- | --- | --- | --- | --- | --- | --- | --- | --- | --- | --- | --- | --- | --- | --- | --- | --- | --- | --- | --- | --- | --- | --- | --- | --- | --- | --- | --- | --- | --- | --- | --- | --- | --- | --- | --- | --- | --- | --- | --- | --- | --- | --- | --- | --- | --- | --- | --- | --- | --- | --- | --- | --- | --- | --- | --- | --- | --- | --- | --- |
|  |  | |  | | **Resistance mutation genotypic profile** | | | | | | | | | | | | | | | | | | | |  | | **Resistance phenotypic profile** | | | | | | | | | | | | | | | | | |  | |  | |  | | |  | |  | |  | |  | |  | |  | | | |  | | | |  |  | | | | |  | | | |  | |  | | | |  | |  |  |
| Patient ID | Sample ID | | Isolate Year | | INH | | RIF | | EMB | | FQ | | STR | | AMK | | CAP | | KAN | | PZA | | ETH | | G-DR-Type | | RIF | | I N H | | P Z A | | E M B | | S T R | | AM I | | O F X | | K A N | | C A P | | Sub-lineage | | *Homo SNPs Call* | | | *Het SNPs call* | | | Sex | | | | Smoker | | Diabetes | | | | | | H I V | | | Lung lesion | | | | | | | Lung lesion | | | | | | Clinical Outcome | | | | | | SNP distance | | |
|  |  |  |  |  |  |  |  |  |  |  |  |  |  |  |  |  |  |  |  |  |  |  |  |  |  |  |  |  |  |  |  |  |  |  |  |  |  |  |  |  |  |  |  |  |  |  |  |  |  |  |  |  |  |  |  |  |  |  |  |  |  |  |  |  |  |  |  | (First available) | | | | | | | (Last available) | | | | | |  |  |  |  |  |  |  |  |  |
| P26 | G23318 | | 2011 | | S315T | | S450L | | M306V | | D94Y | |  | |  | |  | |  | |  | |  | | p-XDR | | R | | R | | - | | R | | - | | - | | - | | - | | - | | L4.3/LAM | | 848 | | | 7 | | |  | | | |  | |  | | | |  | | | | |  | | | | | | |  | | | | | |  | | | | | |  | | |
| P26 | G23323 | | 2011 | | S315T | | S450L | | M306V | | D94Y | |  | |  | |  | |  | |  | |  | | p-XDR | | R | | R | | - | | R | | - | | - | | - | | - | | - | | L4.3/LAM | | 847 | | | 6 | | |  | | | |  | |  | | | |  | | | | |  | | | | | | |  | | | | | |  | | | | | |  | | |
| P26 | G23333 | | 2011 | | S315T | | S450L | | M306V | | D94Y | |  | |  | |  | |  | |  | |  | | p-XDR | | R | | R | | S | | R | | - | | - | | - | | - | | - | | L4.3/LAM | | 849 | | | 7 | | |  | | | |  | |  | | | |  | | | | |  | | | | | | |  | | | | | |  | | | | | |  | | |
| P26 | G23251 | | 2012 | | S315T | | S450L | | M306V | | D94Y | |  | |  | |  | |  | |  | |  | | p-XDR | | R | | R | | S | | R | | - | | - | | - | | - | | - | | L4.3/LAM | | 796 | | | 12 | | | M | | | | No | | No | | | | No | | | | | Bilateral Cavitary | | | | | | | Unilateral Cavitary | | | | | | Death | | | | | | 0 | | |
|  |  | |  | |  | |  | |  | |  | |  | |  | |  | |  | |  | |  | |  | |  | |  | |  | |  | |  | |  | |  | |  | |  | |  | |  | | |  | | |  | | | |  | |  | | | |  | | | | |  | | | | | | |  | | | | | |  | | | | | |  | | |
| P56 | G23164 | | 2012 | | S315T | | S450L | | M306V | |  | |  | |  | |  | |  | |  | |  | | MDR | | R | | R | | S | | S | | - | | - | | - | | - | | - | | L4.3/LAM | | 832 | | | 28 | | |  | | | |  | |  | | | |  | | | | |  | | | | | | |  | | | | | |  | | | | | |  | | |
| P56 | G23208 | | 2012 | | S315T | | S450L | | M306V | |  | |  | |  | |  | |  | |  | |  | | MDR | | R | | R | | S | | S | | - | | - | | - | | - | | - | | L4.3/LAM | | 857 | | | 30 | | | M | | | | No | | No | | | | No | | | | | Bilateral Cavitary | | | | | | | Bilateral Cavitary | | | | | | Cured | | | | | | 0 | | |
|  |  | |  | |  | |  | |  | |  | |  | |  | |  | |  | |  | |  | |  | |  | |  | |  | |  | |  | |  | |  | |  | |  | |  | |  | | |  | | |  | | | |  | |  | | | |  | | | | |  | | | | | | |  | | | | | |  | | | | | |  | | |
| P32 | G23304 | | 2011 | | S315T | | S450L | | M306V | |  | | K43R | |  | |  | |  | |  | |  | | MDR | | R | | R | | - | | S | | R | | - | | - | | - | | - | | L4.10/PGG3 | | 857 | | | 13 | | |  | | | |  | |  | | | |  | | | | |  | | | | | | |  | | | | | |  | | | | | |  | | |
| P32 | G23307 | | 2011 | | S315T | | S450L | | M306V | |  | | K43R | |  | |  | |  | |  | |  | | MDR | | R | | R | | - | | - | | R | | - | | - | | - | | - | | L4.10/PGG3 | | 857 | | | 5 | | |  | | | |  | |  | | | |  | | | | |  | | | | | | |  | | | | | |  | | | | | |  | | |
| P32 | G23311 | | 2011 | | S315T | | S450L | | M306V | |  | | K43R | |  | |  | |  | |  | |  | | MDR | | R | | R | | R | | S | | R | | - | | - | | - | | - | | L4.10/PGG3 | | 855 | | | 10 | | | M | | | | No | | No | | | | No | | | | | Unilateral Cavitary | | | | | | | Unilateral Cavitary | | | | | | Cured | | | | | | 0 | | |
|  |  | |  | |  | |  | |  | |  | |  | |  | |  | |  | |  | |  | |  | |  | |  | |  | |  | |  | |  | |  | |  | |  | |  | |  | | |  | | |  | | | |  | |  | | | |  | | | | |  | | | | | | |  | | | | | |  | | | | | |  | | |
| P42 | G23095 | | 2010 | |  | | S450L | | M306I | | A90V | |  | |  | |  | |  | |  | |  | | p-XDR | | R | | R | | S | | S | | S | | - | | - | | - | | - | | L4.3/LAM | | 831 | | | 14 | | |  | | | |  | |  | | | |  | | | | |  | | | | | | |  | | | | | |  | | | | | |  | | |
| P42 | G23310 | | 2010 | |  | | S450L | | M306I | | A90V | |  | |  | |  | |  | |  | |  | | p-XDR | | R | | R | | - | | S | | S | | S | | R | | S | | S | | L4.3/LAM | | 836 | | | 13 | | |  | | | |  | |  | | | |  | | | | |  | | | | | | |  | | | | | |  | | | | | |  | | |
| P42 | G23328 | | 2011 | |  | | S450L | | M306I | | A90V | |  | |  | |  | |  | |  | |  | | p-XDR | | R | | R | | - | | S | | S | | S | | R | | S | | S | | L4.3/LAM | | 829 | | | 5 | | |  | | | |  | |  | | | |  | | | | |  | | | | | | |  | | | | | |  | | | | | |  | | |
| P42 | G23085 | | 2011 | |  | | S450L | | M306I | | A90V | |  | |  | |  | |  | |  | |  | | p-XDR | | R | | R | | - | | S | | - | | - | | - | | - | | - | | L4.3/LAM | | 832 | | | 23 | | | F | | | | No | | No | | | | No | | | | | Bilateral Cavitary | | | | | | | Bilateral Cavitary | | | | | | Death | | | | | | 0 | | |
|  |  | |  | |  | |  | |  | |  | |  | |  | |  | |  | |  | |  | |  | |  | |  | |  | |  | |  | |  | |  | |  | |  | |  | |  | | |  | | |  | | | |  | |  | | | |  | | | | |  | | | | | | |  | | | | | |  | | | | | |  | | |
| P37 | G23182 | | 2008 | | S315T | | S450L | | G406A | |  | | A906G | |  | |  | |  | |  | |  | | MDR | | R | | R | | S | | - | | R | | S | | S | | - | | - | | L4.3/LAM | | 861 | | | 42 | | |  | | | |  | |  | | | |  | | | | |  | | | | | | |  | | | | | |  | | | | | |  | | |
| P37 | G23220 | | 2008 | | S315T | | S450L | | G406A | |  | | A906G | |  | |  | |  | |  | |  | | MDR | | R | | R | | S | | S | | R | | S | | S | | - | | - | | L4.3/LAM | | 866 | | | 22 | | | F | | | | No | | No | | | | No | | | | | Bilateral Cavitary | | | | | | | Bilateral Cavitary | | | | | | Death | | | | | | 0 | | |
|  |  | |  | |  | |  | |  | |  | |  | |  | |  | |  | |  | |  | |  | |  | |  | |  | |  | |  | |  | |  | |  | |  | |  | |  | | |  | | |  | | | |  | |  | | | |  | | | | |  | | | | | | |  | | | | | |  | | | | | |  | | |
| P38 | G23123 | | 2009 | | S315T | | S450L | | G406A | |  | |  | |  | |  | |  | |  | |  | | MDR | | R | | R | | R | | S | | S | | S | | S | | - | | - | | L4.3/LAM | | 872 | | | 17 | | |  | | | |  | |  | | | |  | | | | |  | | | | | | |  | | | | | |  | | | | | |  | | |
| P38 | G23306 | | 2011 | | S315T | | S450L | | G406A | |  | |  | |  | |  | |  | |  | |  | | MDR | | R | | R | | - | | S | | S | | - | | - | | - | | - | | L4.3/LAM | | 867 | | | 11 | | | M | | | | Yes | | No | | | | No | | | | | Unilateral Cavitary | | | | | | | Bilateral Cavitary | | | | | | Death | | | | | | 0 | | |
|  |  | |  | |  | |  | |  | |  | |  | |  | |  | |  | |  | |  | |  | |  | |  | |  | |  | |  | |  | |  | |  | |  | |  | |  | | |  | | |  | | | |  | |  | | | |  | | | | |  | | | | | | |  | | | | | |  | | | | | |  | | |
| P12 | G23096 | | 2010 | | S315T | | S450L | | G406A | |  | |  | |  | |  | |  | |  | |  | | MDR | | R | | R | | - | | S | | S | | - | | - | | - | | - | | L4.10/PGG3 | | 815 | | | 48 | | |  | | | |  | |  | | | |  | | | | |  | | | | | | |  | | | | | |  | | | | | |  | | |
| P12 | G23314 | | 2011 | | S315T | | S450L | | G406A | |  | |  | |  | |  | |  | |  | |  | | MDR | | R | | R | | S | | S | | - | | S | | R | | S | | S | | L4.10/PGG3 | | 823 | | | 9 | | | F | | | | Yes | | No | | | | No | | | | Bilateral Cavitary | | | | | Bilateral Cavitary | | | | | Cured | | | | | | | | 0 | | | | |
|  |  | |  | |  | |  | |  | |  | |  | |  | |  | |  | |  | |  | |  | |  | |  | |  | |  | |  | |  | |  | |  | |  | |  | |  | | |  | | |  | | | |  | |  | | | |  | | | | |  | | | | |  | | | | | |  | | | | |  | | | | |  |
| P64 | G23100 | | 2010 | | S315T/ C-15T | | S441L | |  | |  | |  | |  | |  | |  | |  | | C-15T | | MDR | | R | | R | | - | | S | | R | | S | | S | | S | | S | | L4.1.2/Harlem | | 776 | | | 26 | | |  | | | |  | |  | | | |  | | | | |  | | | | |  | | | | | |  | | | | |  | | | | |  |
| P64 | G23147 | | 2010 | | S315T/ C-15T | | S441L | |  | |  | |  | |  | |  | |  | |  | | C-15T | | MDR | | R | | R | | S | | S | | - | | S | | S | | R | | R | | L4.1.2/Harlem | | 811 | | | 36 | | | M | | | | No | | No | | | | No | | | | | Bilateral Cavitary | | | | | Bilateral Cavitary | | | | | | Cured | | | | | 0 | | | | |  |
|  | |  | |  | |  | |  | |  | |  | |  | |  | |  | |  | |  | |  | |  | |  | |  | |  | |  | |  | |  | |  | |  | |  | |  | |  | | |  | | | |  | | | | | |  | | |  | | | | |  | | | | |  | | | |  | | | | |  | | | | | | |

**Table S7.** Genotypic and phenotypic profiles of the 82 patients with cured and deceased treatment outcome

| ***n* Samples** | **Genotypic  resistance  classification** | **Phenotypic  resistance  classification** | **Ethambutol  genotypic  resistance profile** | **Ethambutol  phenotypic  resistance profile** | **Outcome** |
| --- | --- | --- | --- | --- | --- |
| 4 | MDR | MDR | R | R | Cured |
| 3 | p-XDR | MDR | R | R | Cured |
| 1 | p-XDR | p-XDR | R | R | Cured |
| 11 | MDR | MDR | R | S | Cured |
| 1 | MDR | p-XDR | R | S | Cured |
| 1 | Poly-R | MDR | R | S | Cured |
| 3 | p-XDR | MDR | R | S | Cured |
| 1 | p-XDR | p-XDR | R | S | Cured |
| 1 | MDR | Poly-R | S | R | Cured |
| 1 | MDR | p-XDR | S | R | Cured |
| 1 | Susceptible | p-XDR | S | R | Cured |
| 1 | IMR | MDR | S | S | Cured |
| 14 | MDR | MDR | S | S | Cured |
| 1 | Poly-R | p-XDR | S | S | Cured |
| 3 | p-XDR | MDR | S | S | Cured |
| 1 | RMR | MDR | S | S | Cured |
| 1 | Susceptible | p-XDR | S | S | Cured |
| 1 | p-XDR | RMR | S | S | Cured |
| 5 | MDR | MDR | R | R | Deceased |
| 8 | p-XDR | MDR | R | R | Deceased |
| 2 | p-XDR | p-XDR | R | R | Deceased |
| 5 | MDR | MDR | R | S | Deceased |
| 1 | Poly-R | MDR | R | S | Deceased |
| 2 | p-XDR | p-XDR | R | S | Deceased |
| 1 | p-XDR | Poly-R | R | S | Deceased |
| 1 | Poly-R | p-XDR | R | S | Deceased |
| 1 | MDR | MDR | R | NA | Deceased |
| 1 | MDR | Poly-R | R | NA | Deceased |
| 1 | MDR | p-XDR | R | NA | Deceased |
| 3 | MDR | MDR | S | S | Deceased |
| 1 | Susceptible | Susceptible | S | S | Deceased |
| 4 | MDR | MDR | R | R | Cured |
| 3 | p-XDR | MDR | R | R | Cured |
| 1 | p-XDR | p-XDR | R | R | Cured |
| 11 | MDR | MDR | R | S | Cured |
| 1 | MDR | p-XDR | R | S | Cured |
| **Genotypic profiles proportion among groups** | | | | | |
| **Ethambutol genotypically resistant (*n*=53)** | | | | | |
| MDR = 51.7% (n=29); Poly-R = 5.6% (n=3); p-XDR = 39.6% (n=21) | | | | | |
| EMB genotypically **resistant** / **Cured** (*n*=25) | | | EMB genotypically **resistant** / **Deceased** (*n*=28) | | |
| MDR = 64% (n=16); Poly-R = 4% (n=1);  p-XDR = 32% (n=8) | | | MDR = 46.4% (n=13); Poly-R = 7.1% (n=2);  p-XDR = 46.4% (n=13) | | |
| **Ethambutol genotypically susceptible (*n*=29)** | | | | | |
| MDR = 65.5% (n=19); Poly-R = 3.4% (n=1); p-XDR = 13.7% (n=4)  RMR = 3.4% (n=1) IMR = 3.4% (n=1); Susceptible = 10.3% (n=3) | | | | | |
| EMB genotypically **susceptible** / **Cured** (*n*=25) | | | EMB genotypically **susceptible** / **Deceased** (*n*=4) | | |
| MDR = 64% (n=16); Poly-R = 4% (n=1); p-XDR = 16% (n=4); RMR = 4% (n=1) IMR = 4% (n=1); Susceptible = 8% (n=2) | | | MDR = 75% (n=3); Susceptible = 25% (n=1) | | |
| **Phenotypic profiles proportion among groups** | | | | | |
| **Ethambutol Phenotypically resistant (*n*=26)** | | | | | |
| MDR = 76.9% (n=20); Poly-R = 3.8% (n=1); p-XDR = 19.2% (n=5) | | | | | |
| EMB phenotypically **resistant** / **Cured** (*n*=11) | | | EMB phenotypically **resistant** / **Deceased** (*n*=15) | | |
| MDR = 63.6% (n=7); Poly-R = 9.1% (n=1);  p-XDR = 27.2% (n=3) | | | MDR = 86.2% (n=13); p-XDR = 13.8% (n=2) | | |
| **Ethambutol Phenotypically susceptible (*n*=53)** | | | | | |
| MDR = 81.1% (n=43); Poly-R = 1.8% (n=1) p-XDR = 13.2% (n=7); RMR = 1.8% (n=1); Susceptible = 1.8% (n=1) | | | | | |
| EMB phenotypically **susceptible** / **Cured** (*n*=39) | | | EMB phenotypically **susceptible** / **Deceased** (*n*=14) | | |
| MDR = 87.1% (n=34)  p-XDR = 10% (n=4); RMR = 2.5% (n=1) | | | MDR = 64.3% (n=9); Poly-R = 7.1% (n=1)  p-XDR =21.3% (n=3); Susceptible = 7.1% (n=1) | | |

EMB = Ethambutol, R = Resistant, S = Susceptible, MDR = Multidrug-resistant, p-XDR = Pre-extensively drug-resistant, Poly-R = Poly drug-resistant, IMR = Isoniazid monoresistant, RMR = Rifampicin monoresistant, NA = not available.
